# Plasminogen missense variants and their involvement in cardiovascular and inflammatory disease

**DOI:** 10.3389/fcvm.2024.1406953

**Published:** 2024-06-25

**Authors:** Teresa Brito-Robinson, Yetunde A. Ayinuola, Victoria A. Ploplis, Francis J. Castellino

**Affiliations:** Department of Chemistry and Biochemistry and the W.M. Keck Center for Transgene Research, University of Notre Dame, Notre Dame, IN, United States

**Keywords:** plasminogen, missense mutations, single nucleotide polymorphisms, plasminogen deficiencies, ligneous conjunctivitis

## Abstract

Human plasminogen (PLG), the zymogen of the fibrinolytic protease, plasmin, is a polymorphic protein with two widely distributed codominant alleles, PLG/Asp^453^ and PLG/Asn^453^. About 15 other missense or non-synonymous single nucleotide polymorphisms (nsSNPs) of PLG show major, yet different, relative abundances in world populations. Although the existence of these relatively abundant allelic variants is generally acknowledged, they are often overlooked or assumed to be non-pathogenic. In fact, at least half of those major variants are classified as having conflicting pathogenicity, and it is unclear if they contribute to different molecular phenotypes. From those, PLG/K^19^E and PLG/A^601^T are examples of two relatively abundant PLG variants that have been associated with PLG deficiencies (PD), but their pathogenic mechanisms are unclear. On the other hand, approximately 50 rare and ultra-rare PLG missense variants have been reported to cause PD as homozygous or compound heterozygous variants, often leading to a debilitating disease known as ligneous conjunctivitis. The true abundance of PD-associated nsSNPs is unknown since they can remain undetected in heterozygous carriers. However, PD variants may also contribute to other diseases. Recently, the ultra-rare autosomal dominant PLG/K^311^E has been found to be causative of hereditary angioedema (HAE) with normal C1 inhibitor. Two other rare pathogenic PLG missense variants, PLG/R^153^G and PLG/V^709^E, appear to affect platelet function and lead to HAE, respectively. Herein, PLG missense variants that are abundant and/or clinically relevant due to association with disease are examined along with their world distribution. Proposed molecular mechanisms are discussed when known or can be reasonably assumed.

## Introduction

Inherited single amino acid substitutions are an important source of potential phenotypic variation between individuals that can lead to disease risk (1, 2) and can contribute to complex multifactorial disorders (3). About one-half of known genetic conditions are caused by nonsynonymous single nucleotide polymorphisms (nsSNPs) (3, 4). Single amino acid mutational studies are limited but naturally-occurring missense variants with associated phenotypes can provide very valuable information for analysis of structure-function relationships of proteins. Techniques such as targeted exome sequencing play key roles in discovering alleles associated with Mendelian and complex disorders.

A comprehensive review of disease-associated PLG missense variants in world populations is highly relevant to more fully comprehend their overall causative influences on coagulopathies and inflammatory diseases and the involvement of the fibrinolytic system in these processes. In this review, we explore the ramifications of naturally occurring variants on the abundant multi-functional soluble plasma protein zymogen, PLG, and its activated product, the serine protease, plasmin. We begin this review with a summary of the background on PLG/plasmin structure-function which is necessary to better understand the mechanisms of the effects of missense variants on the properties of PLG and plasmin.

## Human plasminogen

**Figure 1:**
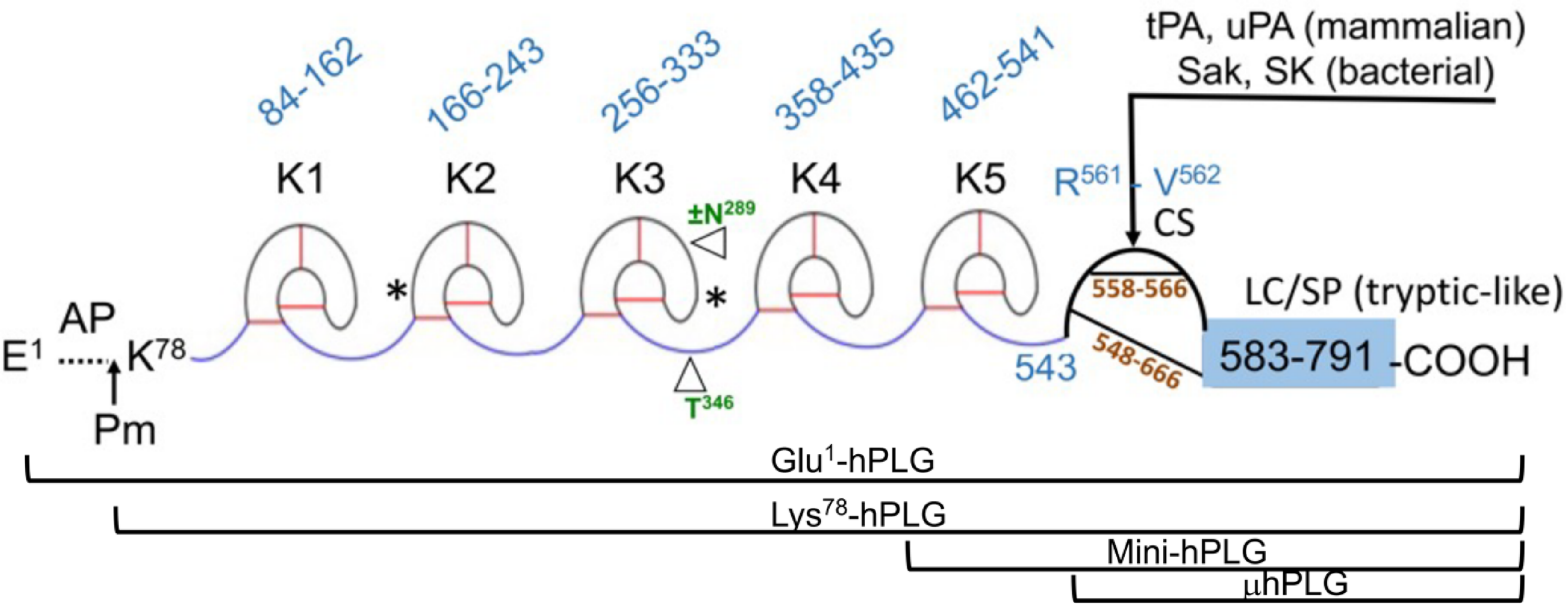
The mature form of the zymogen, human plasminogen (Glu^1^-PLG). After cleavage of the 19-amino acid residue signal sequence, the protein contains 791 amino acids in a single chain. A heavy chain (HC) of 561 amino acids is comprised of five ∼80 amino acid triply disulfide linked kringle (K) domains with inter kringle linker regions (ID). A 229-amino acid light chain (LC) is homologous to serine proteases (SP) such as trypsin. This protease chain is silent in intact PLG but becomes active when PLG activators (PA) catalyze cleavage of the Arg^561^-Val^562^ peptide bond at the cleavage site (CS), providing human plasmin with the LC doubly disulfide-linked to the HC at residues 558/566 and 548/666. The AP is released during this activation process by the generated plasmin. The final plasmin contains residues Lys^78^-Asn^791^ (Lys^78^-PLG) with the HC and LC linked by two disulfide bonds. Note that both the HC and the LC are latent in the zymogen. A single N-linked glycosylation site is present at Asn^289^, which is occupied in ∼60% of the mature protein molecules and a single O-linked glycosylation site at Thr^346^ is occupied in 100% of the mature protein molecules. Other post-translational forms of PLG that occasionally appear in the literature are bracketed below the Figure.

Human plasminogen is encoded by the *PLG* gene, which is located on human chromosome 6q26. The PLG DNA contains 19 exons separated by 18 introns and is 51,861 bp in length (http://genome.ucsc.edu/) (5). PLG is translated primarily in the liver (6), along with minor production in extrahepatic cells. The translated protein is a single-chain 810 amino acid protein without enzymatic activity. Upon maturation, the 19-amino acid signal peptide is removed, and two carbohydrate chains are placed on PLG side-chains, Asn^289^ and Thr^346^ (Figure 1) (7–9), as well as a phosphorylation site of unknown significance located at Ser^578^ (10).

In numbering PLG residues, the fully translated protein, which includes the 19-residue signal peptide, is frequently used in the literature when referring to genomic data and clinical case reports. For example, the fully translated protein numbering for the codominant allelic variant of PLG is written as p.D^472^N, while the corresponding mature protein number is PLG/D^453^N (lacks the signal peptide). In this review, we used the mature protein numbering for all PLG variants in the text. To enhance comparison with data from the literature, the fully translated and the mature protein numbering for PLG variants are stated side-by-side in the Tables.

The mature PLG protein (Glu^1^-PLG) is multi-modular, containing consecutively from the amino terminus (Figure 1): a 77-residue activation peptide (AP), followed by five ∼80 residue triply disulfide-linked kringle (K) domains separated by variable length inter-kringle residues; an activation cleavage site (R^561^-V^562^) susceptible to the catalytic cleavage activity of plasminogen activators (PAs), and a light chain homologous to serine proteases, such as trypsin and chymotrypsin. After direct hydrolysis of the R^561^-V^562^ peptide bond, as catalyzed by PAs, such as urokinase-type plasminogen activator (uPA) and tissue-type plasminogen activator (tPA), or indirect activation by bacterial activators, *e.g.,* streptokinase (SK) and staphylokinase (Sak), the final protease, plasmin (EC 3.4.21.7), is formed. Plasmin consists of the plasmin/[K^78^-R^561^] heavy chain (HC), containing all five kringles, doubly disulfide-linked to the PLG/[V^562^-N^791^] light chain, or serine protease (SP) domain, containing the serine protease catalytic triad, His^603^-Asp^646^-Ser^741^ (11–13). After activation, the resulting plasmin lacks the AP, the removal of which is autocatalyzed by plasmin (14). The HC and the LC are latent in the zymogen (PLG). Also provided in Figure 1 are other derivatives of Glu^1^-PLG, which have occasionally been described in the literature, *e.g.,* mini-PLG and micro-PLG (μPLG), but these are proteolytic products of native PLG, or cloned fragments of this protein, and are not further discussed herein. The post-translational product, Lys^78^-PLG, is an important activation intermediate of Glu^1^-PLG and will be referred to in this review.

## The kringle domains and their lysine binding sites

**Figure 2:**
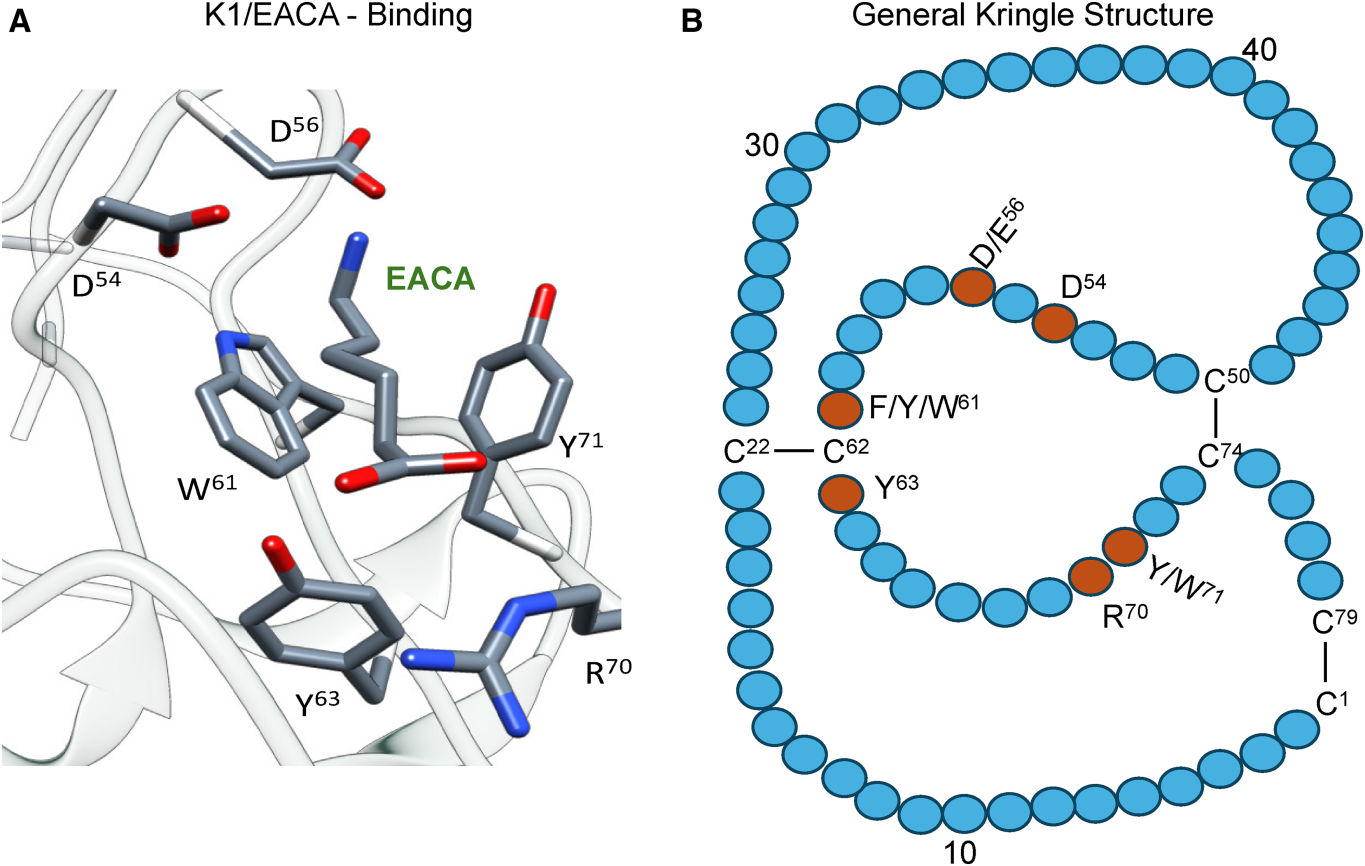
The essential binding residues for a LBS to be present in a kringle. (**A**) The x-ray crystal structure of the binding of a lysine analog, ε-aminocaproic acid (EACA), to isolated PLG-K1(PDB ID, 1CEA). Asp(D)/Glu(E) side chains at residues 54 and 56 (numbering beginning at Cys^1^ of the kringle) are positioned to interact with the ε-amino group of EACA and Arg(R)^70^ bridges the COOH group of EACA. Aromatic residues at amino acids Trp(W)^61^ and Tyr(Y)^71^ stabilize the central methylene groups of EACA. Tyr(Y)^63^ forms a hydrogen bond with the COOH group of EACA. (**B**) A generic 79-residue kringle (based on PLG-K1) is shown emphasizing the locations of the critical amino acids that are needed for strong binding.

Of essential importance to PLG/plasmin function, are the five kringle domains of the PLG-HC, four of which, *viz.,* K1, K2, K4, and K5, bind to lysine with varying affinities**.** Figure 2A shows the x-ray crystal structure of the binding of a lysine analog, ε-aminocaproic acid (EACA), to isolated PLG-K1 and the figure highlights the critical lysine binding residues (15). Figure 2B represents a generic 79-residue lysine binding kringle, based on the numbering in PLG-K1. The location of the important lysine binding residues for each of the kringle modules of PLG is summarized in Table 1. Of course, other residues can assist in the stabilization of the ligand, but the residues shown are important for binding in each of the kringle domains.

**Table 1:** Critical amino acids/centers necessary for the lysine binding function of each kringle domain of PLG. EACA is used as a lysine analog.

K1	K2	K3	K4	K5	Critical center	Mechanism	Generic kringle critical residues
D137	D219	D309	D411	D516	Anionic Center	Coordinates with the ε-amino group of EACA	D54
D139	E221	K311	D413	D518			D/E56
W144	W225	W315	W417	W523	Hydrophobic Center	Stabilizes the central methylene groups of EACA	W61
Y154	W235	W325	W427	Y533			Y/W71
Y146	F227	H317	F419	Y528	Cationic Center	Forms a hydrogen bond with the carboxyl group of EACA	Y/F/H63
R153	R234	R324	R426	L532		Interacts with carboxyl group of EACA	R/L70

K1, K2, K3, K4 and K5: kringle domains of PLG. Amino acids in red font lack the required amino acid at the needed position. These kringles either do not interact with lysine (*e.g.,* PLG-K3) or bind more weakly to EACA than PLG-K1 (*e.g*., K2, K4, or K5).

There are three major centers within the LBS that are essential for the lysine-binding event (Figure 2A). **Firstly,** an anionic center, formed by two aspartates, Asp^54^ and Asp^56^ (numbering beginning at C^1^ of the generic kringle), that coordinate with the amino group side chain of lysine, lysine isosteres, and lysine analogs, such as EACA. Notably, one of these aspartates is replaced by glutamate in PLG-K2 and by lysine in PLG-K3. **Secondly**, a hydrophobic core center, in which two aromatic amino acids, in this case, Trp^61^ and Tyr^71^_,_ form a cluster that stabilizes the central methylene groups of EACA, and lastly, **a cationic center**, composed of basic residue(s), which interact with the carboxylate group of the ligand. As shown in Figure 2A, Arg^70^ interacts with the COOH group of EACA, while Tyr^63^ not only supports the hydrophobic core but also forms a hydrogen bond with the COOH group of EACA. Multiple studies indicate that residue-to-residue variations among the kringle domains, highlighted in Table 1, affect their lysine binding affinities.

Additionally, at least for the binding of EACA to K1-PLG, an Arg at position 34 further stabilizes the carboxyl group of the ligand. Phe^35^ contributes to the hydrophobic cluster that surrounds the backbone of EACA, while the side chains of Tyr^71^ and Tyr^73^ support the anionic center by having interatomic distances that suggest that it can serve as a hydrogen binding partner for EACA and for Asp^56^, respectively, thus stabilizing this latter residue in the lysine binding pocket (Figure 2A). In studies with the isolated kringle domains, PLG-K1 has the highest lysine binding affinity, followed by PLG-K4, PLG-K5 and PLG-K2, while PLG-K3 poorly binds to EACA (16), due to the presence of a lysine (Lys^56^) instead of the acidic amino acid side chain, Asp^56^ (Figure 2B), in its anionic center. Further, PLG-K5 contains Leu^70^, rather than Arg^70^ in its cationic center, a factor that likely governs its weaker binding to EACA (17, 18).

## Functions of LBS in receptor binding and regulation of the PLG conformation

The lysine binding sites (LBS) of kringle domains are critical for the functional properties of PLG and allow PLG and plasmin to bind to cellular receptors utilizing C-terminal lysine residues (19) or internal through-space isosteric lysines formed from proper spacing of amino acid side chains (20). This binding activity stimulates the activation to plasmin and places the potent protease, plasmin, on cell surfaces where it is also resistant to inactivation by natural inhibitors, *e.g.,* α2-antiplasmin (α2AP) and α2-macroglobulin (α2M) (21).

The PLG/plasmin system is primarily involved in the degradation of fibrin but also is a key participant in other proteolytic migratory cellular functions, including tissue repair, extracellular matrix degradation, angiogenesis, tumor invasion, inflammatory cell migration, complement protein interactions, and in maintaining healthy body mucosal surfaces by removing fibrin and misfolded proteins from extravascular tissues (22–27). The PLG activation system is tightly regulated by serpin inhibitors of PAs, such as plasminogen activator inhibitors-1 (PAI-1) and -2 (PAI-2).

Since any free plasmin generated in plasma would be rapidly inactivated by circulating protease inhibitors, most of the pathophysiological cell migratory functions of plasmin*, e.g.,* wound healing, employ cell-bound plasmin. Thus, specific PLG/plasmin cellular receptors are needed. In mammalian cells, glycolytic moonlighting proteins, such as enolase, play important roles in this regard (19, 28–32), whereas in microbial cells, surface proteins, such as M-protein, and even enolase, which migrates from the cytoplasm to the cell surface by an unknown mechanism, are important PLG receptors used by bacteria for migration and dissemination (33).

## PLG closed (T) and open (R) conformations

Not only do the lysine binding sites (LBS) of kringle domains mediate PLG interactions with other proteins, but also basic amino acid side-chains within the AP interact intramolecularly with LBS' of kringle domains, *esp.,* K2-PLG, K4-PLG, and K5-PLG, to place PLG in a tight (T) poorly activatable conformation (34–36). Biochemical and biophysical studies, in addition to the x-ray crystal structure of PLG (37), indicate that the LBS residues of intact PLG, *viz.,* Asp^411^ and Asp^413^ (equivalent to Asp^54^ and Asp^56^ of the isolated PLG-K4), make several interactions with Arg^68^ and Arg^70^ in the AP domain. Additionally, Asp^518^ in the anionic center of the PLG-K5 interacts with Lys^50^ of the AP domain. Likewise, in the LBS of PLG-K2, Asp^219^, Glu^221^, and Arg^234^, interact with other residues located in the SP domain of PLG. These interactions serve to place PLG in a tightly folded and closed activation-resistant T-conformation (32, 38–40), thus maintaining PLG in plasma, which otherwise would be activated, with the resulting plasmin rapidly inactivated by circulating inhibitors. Upon binding to cellular receptors via the LBS, the intramolecular interactions between the LBS' and the AP and SP residues are displaced, inducing a change that relaxes the conformation of the bound PLG (R) rendering it highly activatable (41). This step results in an increased susceptibility of PLG (R) to convert to Lys-PLG by the cleavage of the exposed Arg^560^-Val^561^ peptide bond by plasminogen activators. Most recently, systematic inactivation of critical LBS residues in the various kringle domains of PLG was used to determine their effects on PLG activation by tPA, uPA, or SK. The results indicated that the LBS of PLG-K2 has the highest influence on relaxing the PLG conformation and enhancing its activation potential, followed by PLG-K4 and PLG-K5, with PLG-K1 having the smallest influence (32).

## PLG post-translational variants

Several posttranslational variants of PLG are found in plasma samples and also in purified preparations of the protein. Without inclusion of protease inhibitors in the purification media, a portion of the native Glu^1^-PLG can be converted to Lys^78^-PLG by proteolytic removal of the 77-residue N-terminal AP (Figure 1). The product, Lys^78^-PLG, is far more activatable to Lys^78^-plasmin than is Glu^1^-PLG, but both forms of PLG are converted to the same plasmin, *viz.,* Lys^78^-plasmin (14, 42). Another source of variation in PLG is the two glycoforms separable by specific affinity chromatography on Lysine-Sepharose. This is a general feature of plasminogens from plasmas of different mammalian species (43). These glycoforms have been characterized as a population of PLG not N-glycosylated at Asn^289^ and another form of PLG which is glycosylated with N-linked biantennary complex carbohydrate at Asn^289^. Thr^346^ is fully O-glycosylated in the entire PLG population (7, 8, 44). The properties of these glycoforms have been studied extensively since their discovery and differences between them have been found in lysine binding, PLG activation rates, fibrin binding, and catabolic rates (43, 45–47). In addition, isoelectric focusing (IEF) reveals a number of PLG subspecies ranging in pI from 6.4–8.5 that are primarily derived from differences in sialic acid content on the carbohydrate. Treatment with neuraminidase reduces the number of these bands (45, 48).

Since these variations of PLG are not allelic variants, but are post-translational modification subforms, they will not be further considered in this review.

## PLG polymorphisms

Mutations found in the *PLG* gene include nonsense, missense, frameshift, splice site, deletion and insertion variants that can affect the structure and function of the PLG protein zymogen and its activated product, plasmin. In this review, we focus on missense variants and, to the extent possible, discuss the mechanisms by which these variants can affect the structure and function of PLG and plasmin.

PLG contains several relatively abundant alleles with non-synonymous single nucleotide polymorphisms (nsSNP) that result in missense variants. Some of these alleles appear globally, while others are restricted to different populations. Most of the common PLG missense variants are not thought of being directly deleterious. However, they may contribute via a cumulative effect to increase disease risk when in combination with other PLG variants, or with other protein pathogenic variants and with environmental factors (49, 50). Because PLG plays a critical role in inflammation and disease, it is important to be aware of major PLG variants in the population and their potential effects of PLG/plasmin dysfunction.

The fibrinolytic potential and plasmin generation capacity in individuals can vary significantly and this fact requires attention as to which fibrinolytic drugs should be used in different patients (27). An earlier study reported that the ability to generate plasmin can vary 8-fold in healthy individuals in addition to differences attributed to gender, age, and the use of contraceptives (51). It is not clear whether the existence of polymorphic PLG contributes to some of this variation. The ability to activate PLG to plasmin using different PAs needs to be considered before administering therapeutic treatments to patients carrying certain PLG variants. Understanding the relative world abundance and potential phenotypical consequences of relevant PLG variants is therefore of interest to medicine and population biology, as well as forensics.

## Minor allele frequency (MAF)

In population genetics, the most common allele for a given SNP is referred to as the major allele, while less common alleles are termed minor alleles. The frequency of occurrence of the less common allele (aka, the second-most common allele) is presented as the Minor Allele Frequency (MAF). The MAFs are useful as they provide information about how common a particular SNP is within a given population. The MAF often varies geographically, and both global and regional numbers are important and useful when focusing on populations or resulting protein variants encoded by the allele. Rare alleles are prone to appear locally while common alleles are shared over a wider population range (52).

In this review, MAFs are classified into four groups based on relative abundance ranges:

(1) Polymorphisms: those variants with MAF% ≥5%, corresponding to a MAF ≥0.05.
(2) Common variants with MAF% = 1%–5%, corresponding to a MAF of 0.01–0.05.
(3) Low frequency variants with MAF% = 0.1%–1%, corresponding to a MAF of 0.001–0.01.
(4) Rare and ultra-rare variants with MAF% ≤0.1%, corresponding to a MAF ≤0.001.

While many rare Mendelian diseases are caused by rare (and ultra-rare) variants with large effects, it is believed that both rare and common variants with smaller effects play roles in both complex diseases, but how they work together is unclear (49). Low frequency variants have an important impact in the phenotypic variation at a population scale (53). Genome-wide association studies (GWAS) cannot fully explain the heritability of complex traits (54). This missing heritability effect can be explained by common variants having a weak effect in combination with low-frequency rare variants, which together can lead to complex diseases (55).

## PLG polymorphisms: historical context

The term PLG polymorphisms was first used in the 1970s when researchers started to discover PLG protein variants. During this period, there was no information about MAFs, and PLG polymorphisms were only defined by PLG protein variants carried by the population. Moreover, phenotyping of PLG became of great interest when an abnormal PLG with an unusual electrophoretic mobility pattern was reported in a patient with recurrent thrombosis (56).

Other PLG polymorphisms have since been described using isoelectric focusing (IEF) gel electrophoresis (57, 58). Usually, the procedure to detect PLG polymorphisms by IEF involves treatment of patient plasmas with neuraminidase to remove negatively charged sialic acid from glycan structures and reduce the complexity of the isoforms. The treated plasma is usually next submitted for IEF gel electrophoresis at a pH range 3–10 (or 5–8). PLG is then functionally assayed by activation with uPA or SK with a chromogenic substrate-containing assay kit, and/or by following the lysis of casein in an agar overlay (59). PLG patterns are often obtained by immuno-detection and Western blots.

The interest in PLG polymorphisms increased upon observations that ethnically different populations presented with dissimilar frequencies for certain PLG variants as detected by IEF (60). Accounts of PLG variants in individuals started to accumulate mostly between 1970 and 2000 (56, 61, 62). Some polymorphisms were initially confirmed by amino acid sequencing (63). The PLG/D^453^N polymorphism was identified when the PLG gene was first characterized (5). The phenotypic distribution for the PLG/Asp^453^ and PLG/Asn^453^ alleles was found to fit the Hardy-Weinberg equilibrium, with an autosomal codominant inheritance matching a Mendelian inheritance mode (64).

To identify the many different PLG phenotypes discovered from individual plasmas, an *alpha* numeric nomenclature system was proposed (65). This nomenclature is based on using as a reference the IEF mobility of the two most common PLG polymorphic codominant alleles. They were initially labeled, *PLGA*, with *A* for acidic, and *PLGB*, with *B* for basic. Other alleles were compared to *A* and *B* mobilities in terms of being more *acidic* or more *basic* than these major forms. The identification therefore included *A*-like and *B*-like designations. The letter *M* was used to refer to an intermediate variant or *medium* (between *A* and *B*) and *C* was used for *common* (65). It was soon realized that PLG-based allelic signatures could help generally identify an individual. This gave rise to the use of PLG polymorphisms in forensic hemogenetics, which included paternity examinations (59, 66, 67). It was later found that the polymorphic IEF phenotypes, *PLGA* and *PLGB*, were generated by a single amino acid substitution of the more acidic PLG/Asp^453^ for the relatively more basic PLG/Asn^453^, respectively (68). These polymorphisms were included in many PLG deficiency (PD) case reports and became a reference for the IEF phenotype nomenclature (68).

Prior to standardizing this nomenclature, PLG polymorphisms were difficult to refer to and to compare. Different designations were initially given, including the city of origin of the patient. For example, PLG-Tochigi (69), a mutant with reduced plasmin activity after normal activation, was identified as IEF-M5 and later associated to PLG/A^601^T. PLG-Osaka also produced a PLG variant that led to a form of plasmin with reduced activity (70). This was classified as IEF-M and later identified as PLG/D^676^N. The most frequent IEF patterns often included combinations of one or two wild-type (WT) PLG alleles with a combination of one or two common PLG alleles. Overall, about eighteen phenotypic PLG polymorphisms were initially identified using IEF (71). Other names for variants included PLG-Nagoya (72), PLG-Chicago (73), PLG-Frankfurt (74), and Plasminogen Paris (75). On occasion, phenotypes were identified with designations such as *PLG-1* which was later associated with the *A*-phenotype. Case reports of novel PLG polymorphisms after the year 2000 occasionally use the city of origin of the proband. The PLG-Kanagawa-I polymorphism was reported in 2002 and corresponds to a dysfunctional PLG activity caused by the PLG/G^732^R variant (76).

PLG phenotyping based on the IEF protocol has several advantages, *viz.*: (1) PLG is readily available from patient plasmas for further characterization; (2) the PLG protein band pattern corresponding to the translated alleles from the blood of an individual can be readily visualized; (3) the electrophoretic mobility provides information about the overall charge of the protein as compared to wild-type (WT)-PLG and differences can be an indication of amino acid changes and different alleles; (4) many times an allele is expressed in a lower amount and the relative abundance of alleles could provide phenotypic information; and (5) the isoelectric point for a protein with a known amino acid sequence can be calculated. Since IEF changes may reveal alterations of the PLG structure, the IEF pattern adds valuable information for a phenotypic characterization of a PLG variant in a patient and a first step towards a diagnosis of a PLG deficiency.

The IEF protocol helped to visualize the existence of various PLG phenotypes in plasma and PLG variants sometimes associated with disease. *PLG* genetic analysis was later introduced, especially when young individuals presented unusual symptoms, *e.g.,* thrombosis, which made the search for abnormalities in this gene a valuable approach. The need to purify PLG variants for further analysis was also suggested when subjects were found to carry different IEF patterns (77).

Whereas IEF analysis is still often used as a characterization step, this is usually followed by genomic DNA analysis of *PLG,* including the use of the polymerase chain reaction (PCR), single-strand conformation polymorphism (SSCP) analysis, and/or direct DNA sequencing (68, 78). A summary of several IEF phenotypes with corresponding *PLG* molecular variations has been reported (79).

IEF from case reports of patients and families, combined with DNA sequence information, has contributed to the discovery of many amino acid substitutions in PLG deficiencies. IEF, followed by DNA sequence analysis, was most recently used in the discovery of the PLG/K^311^E missense variant that leads to a rare disease known as hereditary angioedema (HAE) with normal C1 inhibitor. This variants has been cataloged as a clinical variant (80).

## Predictive algorithms of protein dysfunction

Most PLG missense variants of interest lack functional studies and their clinical significance are missing or uncertain. Amino acid variants can range from benign to pathogenic. Predictive *in silico* computational methods can provide highly likely scenarios of amino acid substitutions in proteins, especially when using different approaches (81–83). To facilitate a more comprehensive discussion of the pathogenic variants that will be discussed later in this review, we consider it essential to conduct an *in silico* analysis that predicts potential structural and functional perturbations resulting from various amino acid substitutions in PLG variants. This analysis will enable us to better understand the molecular implications of these variants and provide valuable insights into their pathogenic potential. Herein, we used the following *in silico* prediction tools. SIFT (Sorting Intolerant From Tolerant), which is based on sequence conservation (84); Polyphen-2, which assesses the impact of amino acid substitutions on protein structure/function (85); mCSM, which predicts the effect of variants in proteins using graph-based signatures (86); MUpro, which predicts protein stability changes based on protein sequence and structure and uses Support Vector Machine (SVM) (87); and DynaMut2, which combines Normal Mode Analysis (NMA) methods to capture protein motion and graph-based signatures (88). PLG structural data used for mCSM, MUpro, and DynaMut2 was based on the x-ray structure of Glu^1^-PLG (PDB ID, 4DUR) (36) and the cryo-EM structure of PLG (PDB ID, 8UQ6).

For the amino acid substitution effects using Polyphen-2 and SIFT, the score for substitution of each residue in each prediction tool was first recorded. A red-green heat map was then created from those values by assigning bright red for the most damaging score and bright green for the most tolerated score for each prediction tool. Specifically, Polyphen-2 score 0, benign (green); score 0.5 (mid-range), possibly damaging; score 1, probably damaging (red). SIFT score < 0.05 is predicted to be deleterious (red); score 0, variants can affect protein function (red); and score 1, tolerated (green). We excluded nonsense variants since stop codons cannot be modelled with the prediction software. Clinical variant classifications were based on the ClinVar (NHLBI) algorithm (https://www.ncbi.nlm.nih.gov/clinvar/). Accession numbers for PLG missense clinical variants are provided in the text as appropriate.

The high-resolution structures of PLG have contributed greatly to the understanding of its structure/function relationships and facilitates making credible functional predictions. Studying the effects of single amino acid substitutions in PLG that lead to clinical outcomes, as found in congenital PLG deficiencies, also presents a convenient informational source that can provide critical insights into its role *in vivo*. Animal models, such as PLG gene-altered mice (89, 90), in combination with various other related transgenic murine models, continue to be instrumental in understanding the mechanisms of PLG function.

## Prevalence of PLG missense variants in different populations

In general, population data of a variant is important when evaluating its pathogenicity. Usually, the most abundant variants are not directly pathogenic but may contribute in a minor way to complex diseases, especially if the variant is predicted as pathogenic and if it occurs in a protein like PLG which is involved in many disease mechanisms (50). The chances of a pathogenic condition increase in homozygous or compound heterozygous states where the additive effect increases the penetrance (50).

The gnomAD browser v4.0.0 currently lists ∼1,000 missense *PLG* variants detected from a wide variety of large-scale sequencing projects (https://gnomad.broadinstitute.org/). Most of the *PLG* nsSNPs are rare or ultra-rare (MAF≤ 0.1%), while less than 2% of the variants (Table 2) are relatively abundant (MAF ≥0.1%) in various genetic ancestries in the world. From the 2% group, most major PLG missense variants are assumed to be benign, but in fact, not much is known about them at the molecular level and, therefore, are also of great interest in this review.

**Table 2:** MAF percent distribution of major PLG missense variants per genetic ancestral group (gnomAD database).

Mature protein variant	European (Non-Finnish)	European (Finnish)	East Asian	South Asian	African/African American	Admixed American	Middle Eastern	Ashkenazi Jewish	Amish	Remaining	Total
**K19E**	0.641	0.075	0.000	0.005	0.100	0.243	0.017	0.007	0.000	0.312	0.498
E38K	0.014	0.003	0.000	0.022	4.717	0.410	0.614	0.000	0.000	0.434	0.266
R70K	1.359	0.184	0.002	0.038	0.251	0.283	0.203	0.068	0.000	0.681	1.054
D175V	0.000	0.000	0.697	0.003	0.008	0.017	0.000	0.000	0.000	0.058	0.023
**T181A**	0.142	0.002	0.000	0.000	0.024	0.058	0.000	0.000	0.000	0.090	0.111
**R234H**	0.047	0.000	0.000	0.047	0.015	0.143	0.891	0.324	0.000	0.146	0.058
R242H	0.371	0.547	0.002	0.006	0.072	0.042	0.084	0.000	1.864	0.233	0.307
R389W	0.016	0.002	0.000	0.024	4.040	0.373	0.198	0.000	0.000	0.398	0.231
**G401D**	0.266	0.033	0.000	0.090	0.059	0.150	0.545	0.527	0.000	0.293	0.232
S441R	0.112	0.003	0.000	0.036	4.117	0.617	10.240	0.226	0.000	0.827	0.373
D453N	29.150	26.230	0.067	10.720	17.340	14.980	25.590	29.210	22.310	24.740	25.930
**R471Q**	0.249	0.048	0.000	0.001	0.064	0.022	0.000	0.000	0.000	0.086	0.191
A475V	0.242	0.003	0.136	0.042	1.630	2.671	2.821	5.902	0.000	1.218	0.524
**T481M**	0.004	0.005	0.169	0.011	0.049	0.008	0.000	0.000	0.000	0.014	0.012
R504W	1.521	0.303	0.000	0.021	0.217	0.277	0.099	0.064	3.618	1.000	1.188
A601T	0.002	0.000	1.675	0.021	0.000	0.002	0.000	0.000	0.000	0.029	0.051
**I663N**	0.161	0.005	0.002	0.002	0.032	0.010	0.017	0.003	0.000	0.104	0.124
**G693R**	0.007	0.002	0.000	0.002	0.001	0.003	0.099	0.547	0.000	0.050	0.018

Data adapted from the gnomAD browser v4.0.0. Variants in bolded font (first column) are those that have clinically conflicting, possibly pathogenic, or uncertain significance, while non bolded font variants indicate those considered clinically benign. Yellow: most abundant (polymorphic) variants (≥5 MAF%). Orange: common variants (1–5 MAF%). Blue: low frequency variants (0.1–1 MAF%). Non shaded cells: rare and ultra-rare variants (≤0.1 MAF%). For full protein variants numbering please refer to Tables 4, 6–9.

In addition to the data from gnomAD browser v4.0.0, the data obtained from the PAGE population study (Table 3) are included in this review because they provide access to genomic data from various American populations involving various races and ethnicities that have not been sufficiently represented in a world in which diversity is progressively increasing (91). The multiscale nature of the MAF% distribution of major PLG variants in different ethnic backgrounds is evident in both Tables 2, 3. The PAGE population study compiles allelic data from various populations, including Native Hawaiians and Native Americans, not readily available in the past (91), and studies on a genetic propensity for stroke in such populations can now benefit from these data. As an example, it has been recently reported that these populations have a higher-than-normal propensity to stroke at younger ages with significantly higher stroke mortality in comparison to other regional ancestries in local populations (92).

**Table 3:** MAF percent distribution of major PLG missense variants per genetic ancestry group (PAGE database).

Mature protein variant	South American	Central American	Dominican	Puerto Rican	Cuban	Mexican	African Americans	Asian	Native American	Native Hawaiian	South Asian	Global
D175V	0.00	0.00	0.00	0.01	0.00	0.00	0.02	0.35	0.00	7.72	0.00	0.49
**T181A**	0.05	0.00	0.05	0.04	0.17	0.11	0.02	0.00	0.16	0.09	0.00	0.05
**R242H**	0.00	0.04	0.10	0.05	0.17	0.06	0.09	0.00	0.40	0.04	0.00	0.08
S441R	0.71	0.78	2.98	1.92	1.54	0.44	3.64	0.02	0.95	0.18	0.00	2.05
D453N	14.78	15.43	22.57	21.90	24.63	13.82	18.09	0.16	22.46	7.85	9.80	15.79
**R471Q**	0.10	0.00	0.00	0.03	0.07	0.06	0.07	0.00	0.00	0.07	0.00	0.05
**T481M**	0.00	0.00	0.00	0.01	0.00	0.01	0.05	0.20	0.00	0.02	0.00	0.05
R504W	0.20	0.25	0.39	0.59	0.57	0.22	0.34	0.00	1.19	0.42	0.10	0.34
**A601T**	0.00	0.00	0.03	0.01	0.00	0.00	0.00	1.43	0.00	0.40	0.00	0.18
**G693**R	0.00	0.00	0.00	0.01	0.00	0.00	0.00	0.00	0.16	0.00	0.00	0.01

Data adapted from the PAGE II (2013–2019). Variants in bolded font (first column) are those that have clinically conflicting, possibly pathogenic, or uncertain significance, while non bolded font variants indicate those considered clinically benign. Yellow: most abundant (polymorphic) variants (≥5 MAF%). Orange: common variants (1–5 MAF%). Blue: low frequency variants (0.1–1 MAF%). Non shaded cells: rare and ultra-rare variants (≤0.1 MAF%). For full protein variants numbering please refer to Tables 4, 6–9.

The overall relative distribution trend found with the gnomAD browser for the second minor allele for *PLG* missense variants was consistent with two other populations studies, the ALFA project (https://ncbiinsights.ncbi.nlm.nih.gov/2020/03/26/alfa/), and with the 1000 Genomes project (https://www.ncbi.nlm.nih.gov/bioproject/28889).

**Figure 3:**
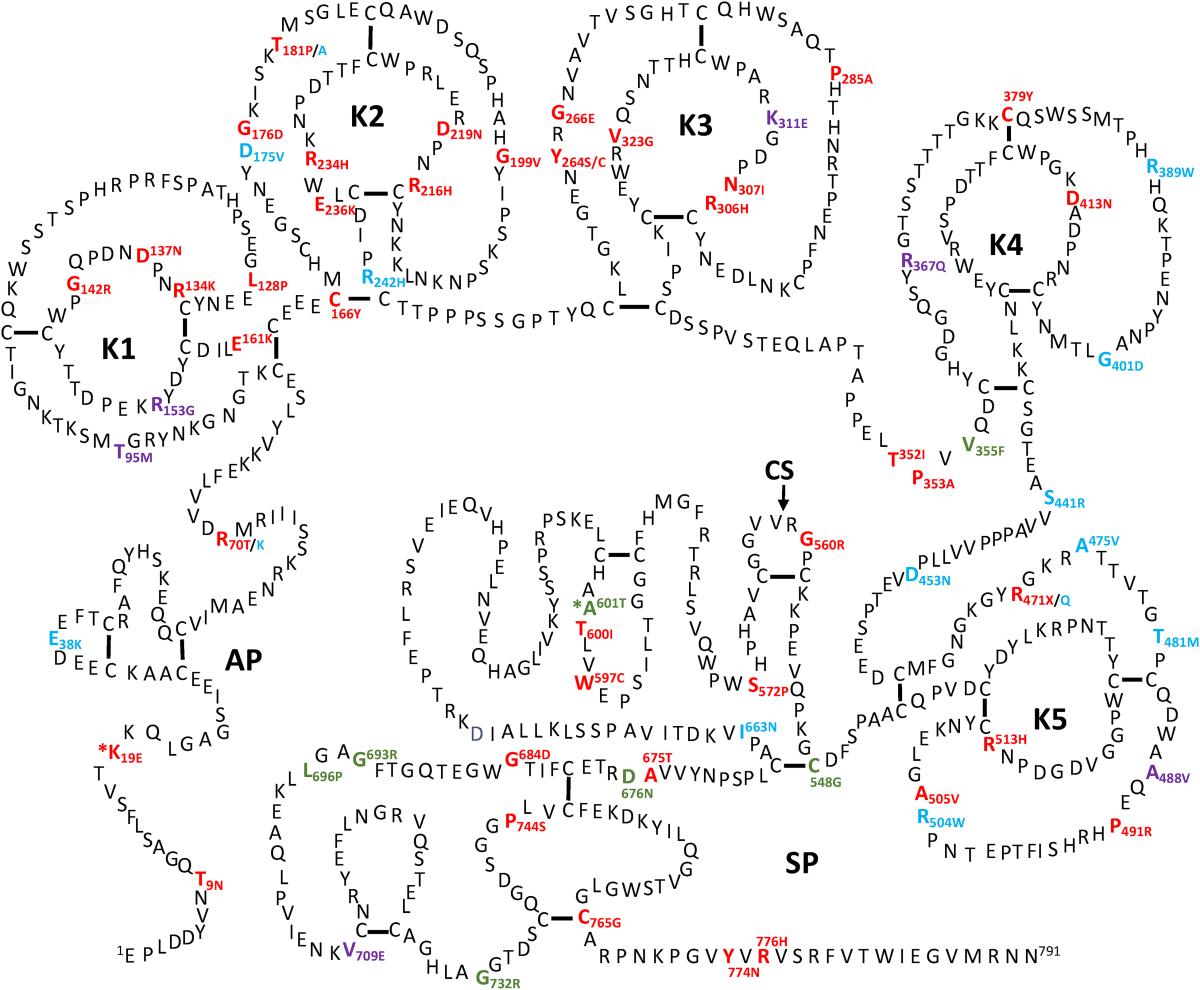
Placement of the known PLG variants within the PLG primary structure. Missense variants are designated red in PDI and green in PDII (green). Major PLG missense variants in the population are presented in blue. Rare pathogenic/or possibly pathogenic PLG missense variants associated with other various disorders (purple). The asterisk in *K^19^E (PDI) and *A^601^T(PDII) indicates that these two variants are also relatively abundant. For full protein numbering of PLG variants refer to the Tables 4, 6–9.

All the PLG missense variants discussed in the present review are illustrated in Figure 3 showing their location along the primary structure of the PLG protein.

## Missense variants and plasminogen deficiency (PD)

PD-associated PLG missense variants have attracted much attention, and they are among the most described in the literature. Except for the relatively abundant PLG/K^19^E and PLG/A^601^T variants, most PD-associated nsSNPs are rare or ultra-rare and are not necessarily always detected in population studies. In fact, most are described in case reports from diseases running within families.

Two types of PD, which include type I (PDI) and type II (PDII), have been described. The PDI and PDII missense variants are represented in red and green font, respectively, in Figure 3. Notably, PLG/K^19^E and PLG/A^601^T are both abundant and related to PD and those have an added asterisk to represent this duality. Single gene Mendelian diseases like PD offer a unique opportunity to study protein structure/function in relationship to disease phenotype.

## PLG deficiency-type I (PDI)

PDI, also known as true PLG deficiency or hypoplasminogenemia, is a genetic disease characterized by low or undetectable PLG antigen. Thus, reduced plasmin activity in plasma is found. This condition results in compromised fibrin clearance (93). Congenital PDI is mostly inherited as an autosomal recessive trait and it is cataloged as a rare disease by the National Organization of Rare Disorders (NORD) (https://rarediseases.org/).

To date, about 45 single amino acid substitutions in PLG have been discovered in probands with PDI. Table 4 summarizes the amino acid substitutions reported for PDI, their domain mapping, and the result of our *in silico* predictions. The current clinical significance for the majority of these substitutions is either missing or has conflicting interpretations.

**Table 4:** PLG missense variants reported in association with PDI with domain locations and predicted sequence-based and structure-based amino acid substitution effects*.

Nucleotide change	Mature protein variant	Full protein variant	Protein domain	Sequence based	Sequence Based	MUpro sequence-based		X-ray PLG structure-based mCSM		Cryo-EM PLG structure-based mCSM		X-ray PLG structure-based DynaMut2		Cryo-EM PLG structure-based DynaMut2		Current clinical classification
				Polyphen-2 score	SIFT score	ΔΔG	Effect	ΔΔG	Effect	ΔΔG	Effect	ΔΔG	Effect	ΔΔG	Effect	
N/A	**L(-11)P**	**p.L8P**	SS	0.999	0.01	-1.96	Destabilizing	N/A	N/A	N/A	N/A	N/A	N/A	N/A	N/A	Not reported
N/A	**T9N**	**p.T28N**	AP	1.000	0.00	-0.63	Destabilizing	−1.51	Destabilizing	−1.23	Destabilizing	−0.97	Destabilizing	−0.77	Destabilizing	Not reported
C.112 A>G	K19E	p.K38E	AP	0.968	0.03	−0.26	Destabilizing	0.27	Stabilizing	0.22	Stabilizing	0.09	Stabilizing	0.03	Stabilizing	Conflicting pathogenicity
c.266 G>C	R70T	p.R89T	AP	0.247	0.00	−1.75	Destabilizing	−1.58	Destabilizing	0.10	Stabilizing	−0.82	Destabilizing	−0.08	Destabilizing	Uncertain significance
C.440 T >C	**L128P**	**p.L147P**	K1	1.000	0.00	−1.71	Destabilizing	−1.65	Destabilizing	−1.66	Destabilizing	−1.38	Destabilizing	−1.33	Destabilizing	Not reported
C.458 G >A	**R134K**	**p.R153K**	K1	1.000	0.00	−1.52	Destabilizing	−1.40	Destabilizing	−1.50	Destabilizing	−2.15	Destabilizing	−1.58	Destabilizing	Not reported
C.466 G >A	**D137N**	**p.D156N**	K1	0.556	0.00	−1.01	Destabilizing	−0.92	Destabilizing	−0.73	Destabilizing	−0.56	Destabilizing	−0.12	Destabilizing	Not reported
C.481 G >A	G142R	p.G161R	K1	0.457	0.00	−0.08	Destabilizing	−1.23	Destabilizing	−1.25	Destabilizing	−0.92	Destabilizing	−0.75	Destabilizing	Not reported
C.538 G >A	E161K	p.E180K	K1	0.017	0.04	−0.91	Destabilizing	−0.60	Destabilizing	−0.31	Destabilizing	−0.03	Destabilizing	−0.13	Destabilizing	Not reported
C.554 G >A	**C166Y**	**p.C185Y**	K2	1.000	0.00	−0.99	Destabilizing	−1.62	Destabilizing	−1.36	Destabilizing	−1.03	Destabilizing	−1.08	Destabilizing	Not reported
C.584 G >A	**G176D**	**p.G195D**	K2	1.000	0.00	−1.33	Destabilizing	−1.12	Destabilizing	−2.03	Highly destabilizing	−0.93	Destabilizing	−1.77	Destabilizing	Not reported
C.598 A >C	T181P	p.T200P	K2	1.000	0.01	−1.50	Destabilizing	−0.54	Destabilizing	0.56	Stabilizing	−0.25	Destabilizing	0.92	Stabilizing	Not reported
C.653 G >T	G199V	p.G218V	K2	0.994	0.18	−0.63	Destabilizing	0.19	Stabilizing	−0.33	Destabilizing	−0.13	Destabilizing	−1.58	Destabilizing	Not reported
C.704 G >A	**R216H**	**p.R235H**	K2	1.000	0.00	−1.44	Destabilizing	−2.02	Highly destabilizing	−2.05	Highly destabilizing	−1.26	Destabilizing	−1.29	Destabilizing	Pathogenic
C.712 G >A	**D219N**	**p.D238N**	K2	0.990	0.08	−0.96	Destabilizing	−0.99	Destabilizing	−1.16	Destabilizing	−0.82	Destabilizing	−0.68	Destabilizing	Risk factor
C.758 G >A	**R234H**	**p.R253H**	K2	0.977	0.01	−1.07	Destabilizing	−1.82	Destabilizing	−1.94	Destabilizing	−0.98	Destabilizing	−1.1	Destabilizing	Likely bening
c.763G >A	**E236K**	**p.E255K**	K2	0.994	0.01	−1.31	Destabilizing	−1.12	Destabilizing	−1.88	Destabilizing	−1.04	Destabilizing	−0.82	Destabilizing	Not reported
C.848 A >C	**Y264S**	**p.Y283S**	K3	1.000	0.00	−1.27	Destabilizing	N/A	N/A	−1.46	Destabilizing	N/A	N/A	−1.02	Destabilizing	Not reported
C.848 A >G	Y264C	p.Y283C	K3	1.000	0.00	−0.98	Destabilizing	N/A	N/A	−0.64	Destabilizing	N/A	N/A	0.25	Stabilizing	Uncertain significance
C.854 G >A	**G266E**	**p.G285E**	K3	1.000	0.00	−0.85	Destabilizing	N/A	N/A	−0.77	Destabilizing	N/A	N/A	−0.49	Destabilizing	Not reported
N/A	**P285A**	**p.P304A**	K3	0.999	0.15	−1.43	Destabilizing	N/A	N/A	−0.60	Destabilizing	N/A	N/A	−0.13	Destabilizing	Not reported
N/A	**P285T**	**p.P304T**	K3	0.998	0.01	−1.34	Destabilizing	N/A	N/A	−0.40	Destabilizing	N/A	N/A	−0.21	Destabilizing	Not reported
C.974 G >A	**R306H**	**p.R325H**	K3	1.000	0.01	−1.44	Destabilizing	N/A	N/A	−2.77	Highly destabilizing	N/A	N/A	−0.67	Destabilizing	Not reported
C.977 A >T	N307I	p.N326I	K3	0.999	0.05	−0.54	Destabilizing	N/A	N/A	−0.07	Destabilizing	N/A	N/A	0.17	Stabilizing	Not reported
C.1026 T >G	**V323G**	**p.V342G**	K3	0.993	0.28	−1.52	Destabilizing	N/A	N/A	−1.55	Destabilizing	N/A	N/A	−1.81	Destabilizing	Not reported
C.1112 C >T	**T352I**	**p.T371I**	ID K3/K4	0.763	0.03	−0.23	Destabilizing	−0.50	Destabilizing	−0.17	Destabilizing	−0.62	Destabilizing	−0.38	Destabilizing	Uncertain significance
C.1114 C >G	P353A	p.P372A	ID K3/K4	0.218	0.33	−0.67	Destabilizing	−1.55	Destabilizing	−0.40	Destabilizing	−1.08	Destabilizing	0.04	Stabilizing	Uncertain significance
C.1193 G >A	**C379Y**	**p.C398Y**	K4	1.000	0.00	−0.30	Destabilizing	−0.10	Destabilizing	−1.00	Destabilizing	−0.29	Destabilizing	−0.87	Destabilizing	Not reported
C.1294 G >A	**D413N**	**p.D432N**	K4	0.414	0.19	−1.54	Destabilizing	−1.11	Destabilizing	−1.17	Destabilizing	−0.75	Destabilizing	−0.85	Destabilizing	Not reported
N/A	R471X	p.R490X	K5	N/A	N/A	N/A	N/A	N/A	N/A	N/A	N/A	N/A	N/A	N/A	N/A	N/A
C.1529 C >G	**P491R**	**p.P510R**	K5	1.000	0.08	−0.91	Destabilizing	−0.3	Destabilizing	−0.90	Destabilizing	−0.07	Destabilizing	−0.41	Destabilizing	Not reported
C.1571 C >T	**A505V**	**p.A524V**	K5	0.994	0.27	−0.69	Destabilizing	−0.6	Destabilizing	−0.21	Destabilizing	−1.08	Destabilizing	−0.86	Destabilizing	Not reported
C.1595 G >A	**R513H**	**p.R532H**	K5	0.958	0.00	−1.44	Destabilizing	−2.3	Highly destabilizing	−1.95	Destabilizing	−0.94	Destabilizing	−1.11	Destabilizing	Not reported
C.1699 T >G	**C548G**	**p.C567G**	SP	1.000	0.04	−2.23	Destabilizing	−1.8	Destabilizing	−2.14	Highly destabilizing	−1.26	Destabilizing	−1.19	Destabilizing	Not reported
c.1735G >A	G560R	p.G579R	SP	0.773	0.62	−0.44	Destabilizing	−1.1	Destabilizing	−0.44	Destabilizing	−0.92	Destabilizing	−0.65	Destabilizing	Uncertain significance
N/A	S572P	p.S591P	SP	1.000	0.07	−1.10	Destabilizing	0.4	Stabilizing	0.30	Stabilizing	1.00	Stabilizing	0.42	Stabilizing	Not reported
C.1848 G >C	**W597C**	**p.W616C**	SP	1.000	0.00	−1.08	Destabilizing	−2.2	Highly destabilizing	−1.52	Destabilizing	−1.81	Destabilizing	−0.91	Destabilizing	Pathogenic
C.1856 C >T	T600I	p.T619I	SP	1.000	0.00	0.19	Stabilizing	−0.4	Destabilizing	−0.39	Destabilizing	−0.72	Destabilizing	−0.76	Destabilizing	Not reprted
c.2080 G >A	A675T	p.A694T	SP	0.094	0.33	−1.31	Destabilizing	−1.4	Destabilizing	−0.77	Destabilizing	−0.92	Destabilizing	−0.06	Destabilizing	Not reprted
C.2108 G >A	**G684D**	**p.G703D**	SP	0.998	0.00	−0.93	Destabilizing	−1.4	Destabilizing	−1.81	Destabilizing	−1.18	Destabilizing	−1.87	Destabilizing	Not reprted
C.2287 C >T	**P744S**	**p.P763S**	SP	1.000	0.00	−1.03	Destabilizing	−2.7	Highly destabilizing	−2.34	Highly destabilizing	−2.77	Destabilizing	−2.01	Destabilizing	Not reprted
C.2350 T >G	C765G	p.C784G	SP	1.000	0.00	−1.08	Destabilizing	−1.0	Destabilizing	−0.75	Destabilizing	−0.15	Destabilizing	0.25	Stabilizing	Not reprted
C.2377 T >A	**Y774N**	**p.Y793N**	SP	1.000	0.00	−0.69	Destabilizing	−1.9	Destabilizing	−1.85	Destabilizing	−1.86	Destabilizing	−1.55	Destabilizing	Not reprted
C.2384 G >A	**R776H**	**p.R795H**	SP	1.000	0.10	−0.72	Destabilizing	−2.0	Highly destabilizing	−1.02	Destabilizing	−1.22	Destabilizing	−1.63	Destabilizing	Not reprted

* Bright green: benign. Bright red: pathogenic. Bolded font variants indicate consistently predicted pathogenicity across the board (light orange shaded cells).

## Clinical manifestations of PDI

The reduced PLG antigen concentration and/or activity characteristic of PDI leads to extravascular accumulation of undigested fibrin and impairs wound healing in mucosal surfaces. This debris causes thick white-yellowish pseudomembranous lesions with a wood-like (ligneous) appearance. Histologically, pseudo-membranes show accumulation of hyaline-like substances, impaired epithelial and fibrin debris with inflammatory cells, fibroblasts, and eosinophilic infiltration (25). The dominant presentation of PDI occurs in the eye-lid surface or conjunctiva, known as ligneous conjunctivitis (LigC), which accounts for up to 80% of the clinical presentations. Similar systemic lesions may occur in additional mucosal tissues, including the gingiva (known as ligneous gingivitis or LigG), the middle ear, the larynx, and the female genital tract. Periodontal disease can be the first clinical manifestation of LigC and PDI (94). Approximately 60% of cases of LigC also develop LigG in PDI with∼a 2:1 ratio of female to male presentation (95). The PLG/K^19^E mutant has been reported in 34% of the LigG cases (95).

Table 5 (Cases A to D) summarizes data from four previously reported case studies involving *PLG* nsSNPs associated with PDI. The data were adapted from case reports and reviews of patients and family members with PDI with no other known health conditions. Notably, data from case reports are often missing critical information. Where available, %PLG activity, %PLG antigen, zygosity, gender, reporting age, and phenotypes are presented. From these available data, PDI symptoms become evident when the %PLG activity is <40%, regardless of the variant. Also, in most cases, homozygotes or compound heterozygotes for certain *PLG* nsSNPs were needed for clinical manifestations of PDI. This is true for PLG/K^19^E, PLG/R^216^H, PLG/W^597^C, and PLG/L^128^P, suggesting an additive effect and different penetrance. PLG/K^19^E may have lower penetrance since at least one homozygous relative did not show clinical manifestations. Variable penetrance and expressivity of variants is a recognized limitation that affects our understanding of the effect of a variant when comparing case reports with the population at large (99). Occasionally, a variant may be sufficiently pathogenic in a given individual to be able to cause clinical symptoms in a heterozygous state, as is the case for PLG/A^505^V variant in Case A, patient 2, in Table 5.

**Table 5A:** Clinical manifestations of PDI missense variants and prediction analysis for several case studies.

Patient	Sex	Allele 1	Allele 2	Polyphen-2 predictions		% PLG activity	PLG antigen mg/dl	Mainly affected tissues/organs	Age of first manisfestation
				Allele 1	Allele 2				
Patient 2	f	A505V	WT	0.994	0.000	45	2.0	Gingiva, Larynx, Vagina	10 years
Patient 8	m	K19E	K19E	0.968	0.968	29	2.0	Conjunctivae, Ears	3 months
Sister		WT	WT	0.000	0.000	49	11.0		
Mother		K19E	WT	0.968	0.000	49	8.0		
Father		WT	K19E	0.000	0.968	41	7.0		
Patient 10	f	K19E	K19E	0.968	0.968	35	4.0	Gingiva	18 years
Sister		K19E	WT	0.968	0.000	51	10.0		
Brother		WT	WT	0.000	0.000	120	15.0		
Brother		K19E	K19E	0.968	0.968	30	5.0		
Father		K19E	WT	0.968	0.000	65	10.0		
Mother		WT	K19E	0.000	0.968	73	11.0		
Patient 14	f	P491R	P491R	1.000	1.000	37	4.0	Conjunctiva	7 years
Father		P491R	WT	1.000	0.000	80	9.0		
Patient 23	m	K19E	K19E	0.968	0.968	27	8.0	Conjunctivae	2 years
Patient 24	f	K19E	K19E	0.968	0.968	33	9.0	Conjunctivae	35 months
Patient 36	f	K19E	WT	0.968	0.000	23	2.0	Gingiva, Ears	6 months
Patient 42	f	R216H	R216H	1.000	1.000	4	<1.0	Conjunctivae, Gingiva	2 months
Father		R216H	WT	1.000	0.000	69	5.0		
Patient 43	f	T181P	T181P	1.000	1.000	25	6.0	Conjunctivae	20 days
Patient 47	n/a	K19E	A505V	0.968	0.994	28	7.0	Conjunctivae	early childhood
Patient 49	f	R216H	R216H	1.000	1.000	2	2.0	Conjunctivae, Duodeno, Bronchus	Birth
Father		R216H	WT	1.000	0.000	40	7.0		
Mother		WT	R216H	0.000	1.000	40	14.0		

**Table 5B:** 

Patient	Sex	Allele 1	Allele 2	Polyphen-2 predictions		% PLG activity	PLG antigen mg/dl	Mainly affected tissues/organs	Age of first manisfestation
				Allele 1	Allele 2				
Patient 1	f	W597C	W597C	1.000	1.000	n.d.	N/A	Conjunctivae	6 years
Patient 2	m	W597C	W597C	1.000	1.000	n.d.	N/A	Conjunctivae	2 years
Patient 4	f	Y264S	K19E	1.000	0.968	13	N/A	Conjunctivae, Vagina	3 weeks
Patient 8	m	K19E	K19E	0.968	0.968	55	N/A	Conjunctivae, Gingiva	2 years
Patient 9	f	K19E	C166Y	0.968	1.000	26	N/A	Conjunctivae	4 years
Patient 10	m	R776H	R776H	1.000	1.000	24	N/A	Conjunctivae, Hydrocephalus	2 years
Patient 11	f	K19E	R216H	0.968	1.000	17	N/A	Conjunctivae, Vagina	7 years
Patient 13	f	R216H	R216H	1.000	1.000	n.d.	N/A	Conjunctivae, Gingiva,Resp. Tract, Hydrocephalus	8 months
Patient 14	f	R216H	K19E	1.000	0.968	n.d.	N/A	Conjunctivae, Gingiva	2 years
Patient 15	f	R216H	K19E	1.000	0.968	41	N/A	Conjunctivae, Vagina	5 years
Patient 16	m	K19E	K19E	0.968	0.968	22	N/A	Conjunctivae, Resp. Tract	2 years

**Table 5C:** 

Patient	Sex	Allele 1	Allele 2	Polyphen-2 predictions		% PLG activity	PLG antigen mg/dl	Mainly affected tissues/organs	Age of first manisfestation
				Allele 1	Allele 2				
Patient 1	f	R216H	R216H	1.000	1.000	6	<0.4	Conjunctivae	16 years
Mother		R216H	WT	1.000	0.000	66	2.0		
Father		R216H	WT	1.000	0.000	60	7.0		
Sister		R216H	WT	1.000	0.000	76	7.5		
Subject	f	WT	W597C	0.000	1.000	66	6.9		

**Table 5D:** 

Patient	Sex	Allele 1	Allele 2	Polyphen-2 predictions		% PLG activity	PLG antigen mg/dl	Mainly affected tissues/organs	Age of first manisfestation
				Allele 1	Allele 2				
Patient 1	m	K19E	R513H	0.968	0.958	17	<0.4	Conjunctivae	5 years
Mother		WT	R513H	0.000	0.958	54	7.0		
Father		K19E	WT	0.968	0.000	88	9.0		
Brother		WT	WT	0.000	0.000	121	15.0		
Patient 2	f	K19E	R216H	0.968	1.000	18	<0.4	Conjunctivae	71 years
Patient 5	m	K19E	L128P	0.968	1.000	17	2.4	Conjunctivae	23 years
Mother		WT	L128P	0.000	1.000	57	7.0		
Father		K19E	WT	0.968	0.000	70	14.0		
Subject H	f	T9N	WT	1.000	0.000	57	7.5		

PolyPhen-2. Bright green: benign. Bright red: pathogenic. (**A**) Adapted from Tefs et al. (96). PLG antigen reference range, 6–25 mg/dl; PLG activity reference range, 70%–140%. (**B**), Adapted from Klammt et al. (78). PLG activity (reference range, 75%–120%). n.d.: not determined in this study. (**C**) Adapted from Schuster et al. (97). PLG antigen (reference range, 6–25 mg/dl); PLG activity (reference range, 80%–120%). (**D**) Adapted from Schuster et al. (98). PLG antigen (reference range, 6–25 mg/dl); PLG activity (reference range, 80% to 120%). For variants numbering please refer to Tables 4, 6–9.

An important finding resulting from case studies of heterozygous relatives of congenital PLG deficiency patients is that PLG activity and PLG antigen concentration can be significantly lower than that considered to be normal, with individuals appearing healthy. As an example, the PLG antigen concentration could go as low as 2 mg/dL, with no disease phenotype reported, when having 66% PLG activity (Table 5, Case C, Patient 1, mother). An estimate of how low the PLG activity can be without disease phenotypes based on these cases is about 50%. When the PLG activity and antigen concentration of PLG are too low, clinical manifestations of quantitative PLG deficiency become evident. Beyond a threshold level, symptomatic patients present extravascular fibrin deposition and systemic inflammation.

Overall, PDI can result in severe consequences, including blindness, tooth loss, and infertility in both males and females (25, 100). This debilitating illness significantly impacts quality of life (101). Furthermore, as a rare disease, PDI poses diagnostic challenges worldwide, resulting in delayed access to limited but potentially life-changing interventions (102).

## Prevalence of PDI

The overall prevalence of congenital PD as homozygous or compound heterozygous is reported to be 1/625,000 (orphanet.net). These numbers are expected to be high within regions where consanguineous unions are common. Notably, about 0.13%–0.42% of the world population can be asymptomatic heterozygous carriers of PD alleles (103). This is an important issue since most PDI-associated variants are predicted to be pathogenic and, as such, they may contribute to disease even in heterozygous individuals. Such pathogenic variants may add to the variation in PLG levels and activities observed in the population and possibly contribute to complex, non-Mendelian diseases.

A significant number of patients with PDI are of Turkish origin (25, 93, 104). Turkey has a 19% consanguineous union frequency, with 58% of those being between first-cousins (105). One report showed that 21 of 50 studied patients were of Turkish origin with consanguineous union between parents in 19 members of that study group (96). The Middle East also presents some of the highest rates of consanguinity in the world, with Arabian first cousin consorts reaching 25%–30% of all marriages (106). Likewise, there is a high rate of common ancestral unions in inner Asia (107) and North African countries (108). Unfortunately, allele databases from these regions are not readily available. There are ongoing efforts to improve the current limited access to genomic data from Lebanon and Africa (109, 110). India is also known to have a high burden of rare recessive genetic diseases (111). Importantly, the most recent version of the gnomAD browser (v4.0.0) now includes Middle Eastern ancestry data, which is known for having high consanguinity.

## Potential mechanisms of PDI

It is not clear how the different PLG missense variants lead to PD and the nature of the molecular mechanisms are equally uncertain. Most of the PLG variants associated with PDI have not been fully characterized beyond IEF. Herein, we utilized prediction software to assess the impact of the resulting substitutions on PLG structure and function. It is believed that the structural changes that occur in PDI variants may result in an impaired secretion and/or a reduction in half-life. Unfortunately, the half-lives of most known PDI variants have not been reported.

**Figure 4:**
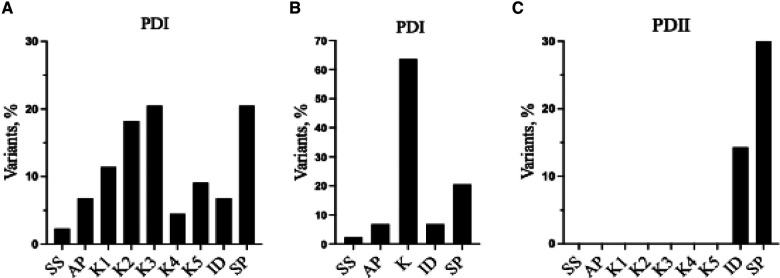
Relative distribution of reported PLG missense variants associated with PDI and PDII in the PLG individual domains. (**A**) PDI variants placed in individual kringles. (**B**) as in (**A**) but merging the PDI variants for all the kringle domains into one kringle (K). (**C**) PDII variants. In (**A**–**C**), ID refers to interdomain.

As indicated by the red-green heat map in Table 4, most PDI substitutions were predicted to be damaging (bright red) for the sequence-based predictions Polyphen-2 and SIFT. The ΔΔG values from the structure-based predictions are consistent with most PDI substitutions being destabilizing when using either protein data base (PDB) structure files. It is seen from this Table that the majority of the PDI variants are located in the very conserved kringle domains of PLG (Column 4, Table 4 and Figures 4A,B) which would destabilize the protein. Interestingly, several PDI variants were consistently predicted to be highly destabilizing. Those variants include PLG/R^216^H in K2-PLG, PLG/R^513^H in K5-PLG, and three variants (PLG/W^597^C, PLG/P^744^S, and PLG/R^776^H) in the SP domain of PLG/plasmin (Table 4). These predictions are consistent with recent findings that show that a functional LBS in K2-PLG is critical to maintain the PLG closed conformation by interactions with the SP domain, and that the LBS’ in K4-PLG and K5-PLG are also critical to maintaining the activation resistant form (32). PDI variants in the SP domain probably mostly destabilize the closed conformation leading to PLG short half-lives. About 20% of PDI substitutions occur in the SP domain (Figure 4B). Herein, we discuss the potential pathogenic mechanisms of some of the variants.

### (A) PLG/K^19^E

The PLG/K^19^E variant is of great interest because it is the most frequent variant that is associated with PDI as a homozygous variant and as a compound heterozygous variant (Table 5A,B,D) and because it is a low frequency variant in several ethnicities (Table 2). For example, the prevalence of PDI due to the PLG/K^19^E allele was reported to be 0.26% for the Scottish population and can be inherited in families (68). This variant is conflictive in terms of prediction tools. It is predicted to be damaging and to affect protein function based on sequence-based predictions (Table 4, Polyphen-2 and SIFT). However, structurally, it seems to be a stabilizing variant (Table 4, mCSM and DynaMut2). This variant has a current clinical status of conflicting pathogenicity in association with PDI and with deep vein thrombosis (VCV000013583.29) and, more recently, this variant has also been associated with *otitis media* (112). The penetrance of PLG/K^19^E is variable, *e.g.,* some heterozygous patients with this variants notably presented gingiva and ear lesions (Table 5, Case A, Patient 36) and another presented periodontal disease and LigC (113). However, other PLG/K^19^E heterozygotes present with a reduction of PLG antigen (lower end of the reference range), but carriers were considered healthy (Table 5, Case A, Patient 8 mother and patient 10 father). Even more conflicting, a homozygous PLG/K^19^E subject (Table 5, Case A, Patient 10 brother) had no symptoms but had a PLG antigen concentration of 5.0 mg/dl, which is below the reference range.

Interestingly, it has been reported that tPA-mediated activation assays could predict the clinical outcome for PLG/K^19^E patients, but uPA-mediated assays could not (114). The uPA-catalyzed activation of PLG is known to be fibrin-independent, whereas the tPA-catalyzed activation is fibrin-dependent (115). It is possible that the PLG/K^19^E variant cannot efficiently engage fibrin, and/or this PLG variant has a short half-life. Lys^19^ is a very conserved residue among primates, and it is also a Lys at this position in mouse PLG. One possible mechanism for PDI resulting from this substitution could be its location in the N-terminus region. In Glu^1^-PLG, this variant may lead to a relaxed conformational state, which is easier to activate and degrade. N-terminus variants, in general, may hinder protein folding during secretion and facilitate access to degrading proteases, which lowers the protein half-life (116). Further analysis is needed to explain the mechanism involved.

### (B) LBS-associated variants

Most variants that lead to PDI are found in the kringle domains (Figures 4A,B) and may affect the integrity of the LBS' of PLG. When LBS' are defective, plasmin may not be able to bind to receptors and substrates and free plasmin will be inactivated by α2AP, which in extravascular tissue will lead to undigested fibrin and other proteins normally cleared by plasmin. The LBS' of PLG are also involved in the proteolytic removal of misfolded proteins (117). Fibrin and misfolded proteins accumulate leading to the mucosal disease observed in LigC*.* With defective LBS', activation by different PAs may also be constricted. PDI mutants that do not occur in the kringle domains, and yet lead to LigC, may follow a different molecular patho-mechanism. Options include substantial protein misfolding, poor secretion, and low half-life. Alternatively, these variants may indirectly damage the ability of kringles to interact with Lysine-containing receptors.

One of the key residues of PLG identified by x-ray crystallography to form important bonds for maintenance of the PLG conformation include Arg^70^ of the AP domain. This residue coordinates with K4-PLG and K5-PLG through interactions with Asp^413^ and Asp^534^ (36). A relatively common PLG missense variant, PLG/R^70^K (Table 2), may still support this conformation since Arg and Lys are basic amino acids. However, substitution of the same residue by Thr in PLG/R^70^T is predicted as possibly damaging (Table 4) and, interestingly, the PLG/R^70^T substitution has been flagged as having a potential association with PDI (RCV001334374.1) and hereditary angioedema (RCV002493731.1). This variant may disrupt the PLG closed conformation with a Thr residue being unable to interact with PLG-K4 and PLG-K5. Thus, this conformation can lead to enhanced PLG activation to plasmin and a subsequent lower half-life of the zymogen.

In a study of the interaction of PLG with Group A *Streptococcus pyogenes* (GAS) surface proteins *in vitro* (32, 118), our group has expressed and characterized the PDI-associated missense variant PLG/D^219^N that affects the K2-LBS anionic ligand binding region. Unlike WT-PLG, PLG/D^219^N precludes GAS from effective invasion by defective interaction with the cell surface PLG binding protein, plasminogen-binding group A streptococcal M-protein (PAM). Carriers of this PDI variants may have a competitive advantage against *S. pyogenes* infection*.* In PLG/D^219^N, the intramolecular salt bridge between K2-PLG and Lys^708^ of the SP residue is disrupted (32, 118). This predicted pathogenic variants would affect the stability of the PLG three-dimensional structure. We found that this variant was easier to activate, most likely due to a more relaxed PLG conformation with increased exposure of the Arg^561^-Val^562^ activation cleavage site (118). This variant should also facilitate proteolysis and reduce the half-life of PLG *in vivo,* which is consistent with PDI.

Other PLG PDI pathogenic variants of the LBS in human disease include PLG/D^413^N (119) and PLG/R^234^H (93) (Table 1). The PLG/D^413^N variant has recently been reported in an adult PDI onset case with severe clinical symptoms in a heterozygous carrier (119). Asp^413^ is an essential residue in the anionic center of the LBS of K4-PLG that forms an important bond necessary for the PLG conformation by coordination with residues Arg^68^ and Arg^70^ in the AP of PLG and with Asp^534^ in K5-PLG (36), retaining PLG in the closed activation-resistant conformation. This patient also developed PLG antibodies that could be the result of the severe conformational change of PLG that became immunogenic. Anti-PLG antibodies in this patient may have triggered the more severe clinical phenotype. The severity of PDI seems to be related to its PLG activity level, the type of the variants, its penetrance, and sometimes a triggering effect, *e.g.,* trauma and infection.

### (C) Impaired secretion

Impaired secretion is one reported mechanism for various PLG missense variants in PDI. The PLG/R^134^K, PLG/R^216^H, PLG/P^285^T, PLG/P^285^A, and PLG/R^776^H variants showed significant secretion impairment and enhanced degradation when they were expressed in monkey COS-7 cells, consistent with their association with PDI (96). Arg^134^ and Arg^216^ are located proximal to strictly conserved Cys residues in the kringle domains and form a network of hydrogen bonds that stabilize the kringle domain. The disruption of the Arg residues in those positions may impair proper folding of the domains and lead to poor secretion. A similar finding has been reported with equivalent missense variants of two Arg residues in the kringle domains of the plasma lipoprotein (a) (homologous to plasminogen) leading to low Lp(a) plasma levels (120). Low plasma levels of Lp(a) are reportedly beneficial in certain cases as they are associated with reduced risk of cardiovascular diseases such as coronary heart disease, peripheral vascular disease, stroke, heart failure and aortic stenosis (121). However, low Lp(a) levels have also been linked to an increased risk of diabetes mellitus and bleeding (122).

## Understanding of PDI mechanism-recommendations

Studying isolated PLG from patient plasmas would be of great interest in order to determine the impacts of critical variants that result in PDI. Activation with SK, uPA, and tPA would also provide important information. Most case reports are limited to overall PLG activation assays using SK and a chromogenic commercial kit, as well as an antigen assay. The method by which PLG activity is measured in patients can affect the conclusions regarding the fibrinolytic potential of PLG variant carriers. The ability to bind fibrin and the activation kinetics with various PAs require clarification. Analysis of PLG variants obtained from homozygous probands should include PLG activation assays with various PAs (123). A functional assay would facilitate defining the molecular mechanism of the disease (114). Measuring fibrinolytic and thrombin generation simultaneously is also a potential strategy.

The manner of measuring fibrinolysis needs reevaluation when PLG deficiencies are reported (124). An important point is that alternative functional PLG/fibrinolysis assays should be considered when evaluating PLG-deficient plasma. A simultaneous test of thrombin and plasmin generation within defined populations may facilitate obtaining a more comprehensive sense of hemostasis regulation among individuals and this strategy has been proposed for a more comprehensive assessment of PD patients and their clinical outcomes (125). This approach was recently used to compare hemostasis among different species, and it was found that using a tPA activation assay of PLG is a much more sensitive and reliable method for plasmin generation than measurement of clot lysis times (126).

## PLG deficiency-type II (PDII)

PDII, also known as dysplasminogenemia is characterized by normal or slightly lower PLG antigen level with a diminished or abnormal activity. The East Asian abundant PLG/A^601^T variant and six other rare and ultra-rare PLG missense variants have been associated with dysplasminogenemia (Table 6). The PLG/A^601^T variant is inherited as an autosomal codominant trait. It circulates in a heterozygous form in otherwise healthy subjects and can lead to a reduced plasmin activity of 14% as a homozygous variant and 57% in heterozygous individuals. It has been proposed that carrying this allele could increase the risk of thrombosis when combined with other factors. Approximately 86% of the reported missense variants associated with PDII are clustered along the serine protease domain, possibly causing disruptions to the catalytic site (Figures 3, 4C).

**Table 6:** PLG missense variants reported in association with PDII with domain location and predicted sequence-based and structure-based amino acid substitution effects*.

				Sequence based	Sequence based	MUpro sequence-based		X-ray PLG structure-based mCSM		Cryo-EM PLG structure-based mCSM		X-ray PLG structure-based DynaMut2		Cryo-EM PLG structure-based DynaMut2		
Nucleotide change	Mature protein variant	Full protein variant	Protein domain	Polyphen-2 score	SIFT score	ΔΔG	Effect	ΔΔG	Effect	ΔΔG	Effect	ΔΔG	Effect	ΔΔG	Effect	Current clinical classification
c.1120G>T	**V355F**	**p.V374F**	ID K3/K4	0.828	0.52	-0.36	Destabilizing	-1.12	Destabilizing	-0.72	Destabilizing	-0.70	Destabilizing	-0.39	Destabilizing	Pathogenic
c.1699T>G	**C548G**	**p.C567G**	SP	1.000	0.04	-2.23	Destabilizing	-1.85	Destabilizing	-2.14	Highly destabilizing	-1.26	Destabilizing	-1.19	Destabilizing	Non reported
c.1858G>A	**A601T**	**p.A620T**	SP	1.000	0.00	-0.34	Destabilizing	-1.44	Destabilizing	-1.76	Destabilizing	-1.42	Destabilizing	-1.67	Destabilizing	Conflicting Pathogenicity
c.2083G>A	D676N	p.D695N	SP	0.000	0.26	-1.39	Destabilizing	-0.70	Destabilizing	-0.33	Destabilizing	-0.62	Destabilizing	-0.67	Destabilizing	Non reported
C.2134G>A	**G693R**	**p.G712R**	SP	0.996	0.34	0.11	Destabilizing	-0.29	Destabilizing	-0.34	Destabilizing	-0.35	Destabilizing	-0.33	Destabilizing	Uncertain significance
c.2144T>C	**L696P**	**p.L715P**	SP	1.000	0.02	-2.15	Destabilizing	-1.57	Destabilizing	-1.33	Destabilizing	-1.17	Destabilizing	-0.35	Destabilizing	Not reported
c.2251G>A	G732R	p.G751R	SP	0.993	0.53	-1.12	Destabilizing	-0.18	Destabilizing	-0.43	Destabilizing	0.02	Stabilizing	-0.30	Destabilizing	Uncertain significance

* Bright green: benign. Bright red: pathogenic. Bolded font variants indicate consistently predicted pathogenicity across the board (light orange shaded cells).

## Clinical manifestations of PDII

Dysplasminogenemias are generally considered to be disorders caused by PLG variants but may not necessarily lead to disease or thrombotic risk. Others believe that this condition can be a risk factor when individuals are challenged by trauma, infection, and environmental influences. An interesting and unexplained observation is that the typical mucosal lesions observed with PDI have never been reported for PDII in either homozygous or compound heterozygous PDII patients (98). The most surprising observation is the apparent lack of deep vein thrombosis (DVT) in most PDII patients. The reason for this may be due to the fact that most PDII associated missense variants have intact kringle domain residues which allow PLG to bind to extravascular receptors. It is hypothesized that mild dysfunction of plasmin activity may suffice to support fibrin degradation. Another hypothesis, supported by previous findings, is that upon binding to receptors, a more functional PLG conformation is favored that may compensate for a dysfunctional activity (41). In a clinical case report, a PLG activity as low as 7.7% was reported for a combined heterozygous Japanese individual carrying PLG/A^601^T and PLG/G^732^R, *viz.,* the Kanagawa-I PLG phenotype (76). The patient had a healthy lifespan, but senile dementia developed at age ≥70, possibly due to unconfirmed multiple cerebral strokes (76). It is hypothesized that low or dysfunctional plasmin activity represents an increased risk for small vessel circulatory diseases. PDI and PDII are both reportedly associated with circulatory abnormalities, including small optic thrombotic retinopathy, ischemic optic neuropathy, and occlusion of the central retinal artery or vein (127). The PLG/A^601^T missense variant was reported in three individuals with retinochoroidal vascular disorder, which presented with low antigen and low PLG activity, but a more severe macular choroidal occlusion occurred when the homozygous disorder was present (127). Recently, a 34-year-old Korean patient heterozygous for PLG/A^601^T presented with a history of recurrent thrombotic arterial embolism and cardiac myxoma. The proband's mother, homozygous for PLG/A^601^T, had a 75% decrease in plasmin activity, but surprisingly had no history of DVT. This lack of correlation of the homozygous variants with thrombotic disease is unclear (128). The PLG/A^601^T variant is, therefore, still classified as a variant of conflicting pathogenicity (VCV000013574.7).

## Prevalence and possible mechanisms of PDII

PLG/A^601^T is the most reported PLG missense variant associated with dysplasminogenemia and is present in up to 4% of East Asian individuals (129). It is associated with the PLG phenotypes of Tochigi I and II, Kagoshima, and Nagoya. This variant is abundant in the Chinese Han population (130), in Korea (131), and in Japan (132). It is also found to be a low-frequency variant of concern in Native Hawaiians**.** The high frequency of PLG/A^601^T in East Asians is believed to have occurred by a founder effect (131).

The mechanisms behind PDII variants may be mostly related to a reduced ability to activate PLG to an effective serine protease. PLG residue Ala^601^ is strongly conserved through multiple species. The molecular mechanism behind the Ala^601^Thr variants has been proposed to involve the nearby His^603^ of the serine protease catalytic triad, which becomes unable to serve as an effective proton acceptor (133). The mouse PLG/A^603^T model, equivalent to the human PLG/A^601^T Tochigi phenotype, showed significantly reduced Pm plasmin activity (8%) after activation by uPA, but did not show a significant difference in a brain ischemia model (129), strongly suggesting that other factors are needed to induce thrombosis. For the PDII-associated PLG missense variant PLG/D^676^N, it was proposed that this variant, which is associated with the PLG-Osaka (IEF *M*), may render PLG inactive because it produces a new N-glycosylation site at the tripeptide, Asn^676^-Arg^677^-Thr^678^, which would disrupt the protease active site (70).

## Understanding of PDII mechanism-recommendations

It has been proposed that dysplasminogenemias should be characterized using highly purified PLG from the plasmas of patients (77). It was argued that the unaccounted existence of SK antibodies, PA inhibitors, and plasmin inhibitors in plasma affect plasmin generation in patients. It was also recognized that patients may be homozygous or heterozygous for PLG variants, which would add extra complexity to phenotypes. It was further proposed to study the binding of SK to the purified PLG, the equimolar PLG-SK complex formation rate, and the active site generation in the complex. For example, PLG may activate with uPA but not with SK. Even purified PLG may contain inactive and active proteins. In those cases, it is important to either confirm with a homozygous protein or to model the variants *in vitro*. This challenge was recognized early and a classification of PLG variants was suggested using twelve different PLG deficiencies that were sorted by classifying active site-deficient PLG. Overall, a further understanding of how the different variants perform will require isolating the variants or expressing them *in vitro*.

## Thrombosis risk in PD

Although elevated PAI-1 and thrombin-activatable fibrinolysis inhibitor (TAFI) are major risk factors for venous thrombosis, the PLG level continues be a potential underlying risk (134). The role of PD-associated variants in contributing to thrombosis is unclear. There are no currently recommended routine genetic determinations for *PLG* polymorphisms to help determine risk for venous or arterial thrombosis. While thrombosis has been observed in PLG-deficient individuals (135), it has mostly been reported in isolated cases within families. Often, an individual nsSNPs is directly paired to a phenotype by clinicians attempting to make a connection between single variants and the *in vivo* PLG mechanism. Such reports also offer a glimpse of their variable penetrance. However, case reports, while providing valuable first analyses, need to be considered with caution because they may be compounded with unknown genetic and environmental factors (136). Such reports need to be supported with other data including those derived from predictive tools, population studies, and functional analysis.

A study in a Japanese population with low PLG activity did not show a significant relationship with thrombosis risk as compared to those with normal PLG activity (137). However, the deficiency was not uniquely characterized in each person, and they were assumed to all have the PLG/A^601^T variant. In a single case report, an individual heterozygous for PLG/A^601^T presented with pulmonary embolism (138). This heterozygous patient only had a 59% decreased PLG activity with no other obvious risk factors, yet this individual required lifelong treatment. In another study, PD could not be excluded as a risk for thromboembolism since no other known or testable factors were present (123). A further report describes a 21-year-old individual with a combined homozygous protein C (PC) deficiency (although it seems unreasonable that this patient would have survived the neonatal period with a total PC deficiency), along with a heterozygous *PLG* variants, presented with recurrent DVT (139). Most recently, two young males (ages 14 and 16) and a young female (age 19) presenting with cerebral infarction were all found to have a significantly reduced PLG activity (∼50%–60%), likely caused by an inherited PLG/A^601^T heterozygous variant. These recent findings continue to support an association of PD with an increased risk of thrombosis. Thrombotic disease is a complex multi-factorial disorder (140) and the role of age, gender, and ethnic background on venous thromboembolism has been recognized and discussed elsewhere (141).

Important contributing factors for the uncertainties of thrombotic disease and PLG deficiencies include the limited number of patients, the diversity of the PLG variants, the variable penetrance, the involvement of different tissues, and the lack of systematic guidelines for reporting the condition. These issues are being addressed by the HISTORY project (102). An important issue is the possibility that PD patients carrying abnormal PLG may be at higher risk of not responding well to classic therapeutic fibrinolytic agents, such as tPA, if a pro-coagulant state is present (27). Heterozygous carriers of abnormal PLG(s) may be more susceptible to incorrect therapeutics due to lack of risk awareness.

Studies investigating the correlation between PD and thrombosis risk are hampered by limited sample sizes, primarily due to the rarity of PD as a recessive disease, making it challenging to get enough participants. A study of 9,611 blood donors in Scotland (142) and a study in Japan (137) indicate a wide variation in PLG levels and activities within these populations. Thus, it is difficult to correlate PLG levels/activity with thrombosis. Moreover, these findings imply that defining a universal normal PLG concentration/activity may be challenging, necessitating a reevaluation of the criteria used to define PLG concentration/activity in healthy patients. For example, there could be a spectrum of functional PLG activation potential in different individuals and ethnicities, PLG may be in excess in many individuals, and/or there may be evolutionary conserved compensatory mechanisms for low PLG concentration/activity. Regardless, the physiological consequences of very low PLG antigen and/or plasmin activity in the vasculature or extravascular tissue nevertheless represent a potential risk factor for disease.

## PLG concentrations in normal individuals

Systematic studies reporting plasma PLG concentrations and activities in different human populations are very limited. It is pertinent to reexamine the normal PLG plasma concentration and activity range. Most reports have included a small number of healthy blood donors from a few geographical regions. A PLG antigen concentration average of 16.0 mg/dl (range: 12.3–19.7 mg/dl) was obtained from 100 donors in a study in England (143). While a PLG antigen concentration average of 12.2 mg/dl (range: 7.7–16.8 mg/dl) and a PLG activity of 96.3% (range: 65.9%–126.8%) was reported from 43 blood donors in Hamburg, Germany (144). A review that same year on PLG proposes 20 mg/dl as the normal plasma concentration for Glu^1^-PLG (145). In the Scotland study earlier mentioned, the 9,611 participants age were between 15 and 65, and the PLG level ranged from 9.0–15.0 mg/dl (average, 12 mg/dl), consistent with the∼2-fold range found previously, but the PLG activity showed a surprising 8-fold range variation (20%–200%) in otherwise healthy individuals (51). Another study reported a normal PLG activity ranging between 75 and 120% (78). Moreover, an investigation of PLG activity from 4,517 normal donors from Japan (ages 32–89), prompted by the fact that the Japanese population have an increased tendency to carry the PLG/A^601^T variant, reported a∼4-fold variation in %PLG activity (ranging from 42%–160%) (61, 137).

The reasons for the wide variations in PLG concentration/activity are not fully understood and studies on the factors that determine plasma levels of PLG are also limited (134). The heritability of PLG levels and activity in plasma is not fully understood and has not been addressed systematically in different populations. More recently, GWAS with a cohort of 2,304 young healthy individuals in Ireland (Trinity Student Study) and a group of 507 siblings at the University of Michigan, indicated a heritability factor of up to ∼50% for plasma PLG levels (146). In that study, a relatively abundant PLG missense variant, PLG/R^504^W, was found to be strongly associated with reduced PLG levels (∼13% reduction per allele). Nevertheless, the molecular nature of this PLG mutant has not been addressed. On the other hand, factors found to increase plasma PLG levels in normal individuals included tobacco smoking, female gender, and the use of oral contraceptives (146). The potential phenotypic impact of PLG/R^504^W, and other abundant PLG missense variants in the population, is uncertain. Such PLG variants may further contribute to variations in the fibrinolytic potential among individuals and influence multifactorial disorders.

## PLG missense variants and other disorders

Additional evidence suggests that nsSNPs in PLG gene generate variants that contribute to a range of disorders beyond PD. Moreover, various PD associated variants have also been linked to other diseases. Pathogenic missense variants associated with a specific disease can help identify causal genes (147).

PLG is often a target in GWAS due to its multiple roles in hemostasis. The use of targeted gene panels, or segregation with disease, has allowed the discovery of the association of PLG with several traits and disorders. These include, but are not limited to, bleeding, thrombosis, platelet conditions (148, 149), susceptibility to infection (150), coronary artery disease, periodontitis (151), quantitative trait loci relevant for absorption, distribution metabolism, excretion of drugs in human liver (152), giant cell arteritis (153) and plasma Lp(a) levels (152).

PLG bound to a variety of cell surface receptors is involved in various cellular responses, such as fibrinolysis, cell migration, wound healing, inflammation, and angiogenesis. Therefore, it is not surprising that PD associated variants and other PLG pathogenic missense variants may contribute to other diseases.

## PLG missense variants and other diseases

In this section, we will discuss specific PLG variants and their contributions to diseases in addition to PD. The first group **(A to F)** below includes rare or ultra-rare PLG missense variants (Tables 4, 7) and the second group (**G to K**) below includes relatively abundant PLG missense variants in the population (Tables 2, 3, 8).

**Table 7:** Rare PLG missense variants associated with disorders other than PD and their predicted amino acid substitutions effects* for several *in silico* prediction tools.

				Sequence based	Sequence based	MUpro sequence-based		X-ray PLG structure-based mCSM		Cryo-EM PLG structure-based mCSM		X-ray PLG structure-based DynaMut2		Cryo-EM PLG structure-based DynaMut2		
Nucleotide change	Mature Protein variant	Full protein variant	Protein domain	Polyphen-2 score	SIFT score	ΔΔG	Effect	ΔΔG	Effect	ΔΔG	Effect	ΔΔG	Effect	ΔΔG	Effect	Current clinical classification
c.341C>T	T95M	p.T114M	K1	1.00	0.01	−0.42	Destabilizing	−0.11	Destabilizing	0.01	Stabilizing	0.24	Stabilizing	0.30	Stabilizing	N/A
c.514A>G	**R153G**	**p.R172G**	K1	1.00	0.03	−1.42	Destabilizing	−1.54	Destabilizing	−1.97	Destabilizing	−1.47	Destabilizing	−1.33	Destabilizing	Uncertain significance
c.988A>G	K311E	p.K330E	K3	0.00	1.00	−0.40	Destabilizing	N/A	N/A	−1.00	Destabilizing	N/A	N/A	−0.36	Destabilizing	Pathogenic
c.1157G>A	**R367Q**	**p.R386Q**	K4	1.00	0.07	−0.91	Destabilizing	−0.28	Destabilizing	−0.14	Destabilizing	−0.78	Destabilizing	−0.69	Destabilizing	Uncertain Significance
c.1520C>T	**A488V**	**p.A507V**	K5	0.43	0.04	−0.18	Destabilizing	−0.48	Destabilizing	−0.70	Destabilizing	−0.86	Destabilizing	−0.55	Destabilizing	Uncertain Significance
c.2183T>A	**V709E**	**p.V728E**	SP	1.00	0.37	−1.00	Destabilizing	−2.19	Highly destabilizing	−1.52	Destabilizing	−1.42	Destabilizing	−1.31	Destabilizing	Pathogenic

* Bright green: benign. Bright red: pathogenic. Bolded font variants indicate consistently predicted pathogenicity across the board (light orange shaded cells).

**Table 8:** Major PLG missense variants in the world population with protein domain location and predicted sequence-based and structure-based amino acid substitution effects*.

				Sequence based	Sequence based	MUpro sequence-based		X-ray PLG structure-based mCSM		Cryo-EM PLG structure-based mCSM		X-ray PLG structure-based DynaMut2		Cryo-EM PLG structure-based DynaMut2		
Nucleotide change	Mature protein variant	Full protein variant	Protein domain	Polyphen-2 score	SIFT score	ΔΔG	Effect	ΔΔG	Effect	ΔΔG	Effect	ΔΔG	Effect	ΔΔG	Effect	Current clinical classification
C.112A>G	K19E	p.K38E	AP	0.968	0.03	−0.26	Destabilizing	0.27	Stabilizing	0.22	Stabilizing	0.09	Stabilizing	0.03	Stabilizing	Conflicting Pathogenicity
c.169G>A	E38K	p.E57K	AP	0.002	0.06	−0.95	Destabilizing	0.39	Stabilizing	0.43	Stabilizing	0.29	Stabilizing	0.44	Stabilizing	Benign
c.266G>A	**R70K**	**p.R89K**	AP	0.590	0.00	−1.77	Destabilizing	−1.21	Destabilizing	−0.14	Destabilizing	−1.31	Destabilizing	−0.84	Destabilizing	Urcentain significance
c.581A>T	D175V	p.D194V	K2	0.000	0.29	−0.86	Destabilizing	0.07	Stabilizing	−0.19	Destabilizing	0.29	Stabilizing	−0.29	Destabilizing	Benign
c.598A>G	T181A	p.T200A	K2	0.998	0.11	−1.28	Destabilizing	−0.54	Destabilizing	0.22	Stabilizing	−0.33	Destabilizing	0.39	Stabilizing	Uncertain Significance
c.758G>A	**R234H**	**p.R253H**	K2	0.977	0.01	−1.07	Destabilizing	−1.82	Destabilizing	−1.94	Destabilizing	−0.98	Destabilizing	−1.10	Destabilizing	Likely Benign
c.782G>A	**R242H**	**p.R261H**	K2	0.999	0.11	−0.73	Destabilizing	−1.01	Destabilizing	−0.57	Destabilizing	−1.52	Destabilizing	−0.61	Destabilizing	Conflicting Pathogenicity
c.1222C>T	R389W	p.R408W	K4	0.004	0.09	−0.77	Destabilizing	0.22	Stabilizing	0.20	Stabilizing	0.35	Stabilizing	0.31	Stabilizing	Benign
c.1259G>A	G401D	p.G420D	K4	0.035	0.95	−0.86	Destabilizing	−0.95	Destabilizing	−0.33	Destabilizing	−0.83	Destabilizing	−0.28	Destabilizing	Conflicting Pathogenicity
c.1380T>A	S441R	p.S460R	ID K4/K5	0.146	0.11	−0.92	Destabilizing	N/A	N/A	−0.28	Destabilizing	N/A	N/A	−0.06	Destabilizing	Benign
c.1414G>A	D453N	p.D472N	ID K4/K6	0.000	0.62	−0.75	Destabilizing	N/A	N/A	0.10	Stabilizing	N/A	N/A	−0.10	Destabilizing	Benign
c.1469G>A	**R471Q**	**p.R490Q**	K5	1.000	0.18	−0.43	Destabilizing	−0.26	Destabilizing	−0.09	Destabilizing	−0.67	Destabilizing	−0.64	Destabilizing	Conflicting Pathogenicity
c.1481C>T	**A475V**	**p.A494V**	K5	0.994	0.01	−0.33	Destabilizing	−0.56	Destabilizing	−0.75	Destabilizing	−1.24	Destabilizing	−0.89	Destabilizing	Benign
c.1499C>T	T481M	p.T500M	K5	0.325	0.10	−0.05	Destabilizing	0.43	Stabilizing	0.66	Stabilizing	0.93	Stabilizing	1.02	Stabilizing	Conflicting Pathogenicity
c.1567C>T	R504W	p.R523W	K5	0.037	0.15	−0.44	Destabilizing	−0.32	Destabilizing	−0.44	Destabilizing	−0.6	Destabilizing	−0.44	Destabilizing	Likely Benign
c.1858G>A	**A601T**	**p.A620T**	SP	1.000	0.00	−0.75	Destabilizing	−1.44	Destabilizing	−1.76	Destabilizing	−1.42	Destabilizing	−1.67	Destabilizing	Conflicting Pathogenicity
c.2045T>A	**I663N**	**p.I682N**	SP	0.999	0.40	−0.99	Destabilizing	−2.42	Highly Destabilizing	−2.44	Highly Destabilizing	−1.92	Destabilizing	−1.72	Destabilizing	Conflicting Pathogenicity
C.2134G>A	G693R	p.G712R	SP	0.996	0.34	0.11	Stabilizing	−0.29	Destabilizing	−0.34	Destabilizing	−0.35	Destabilizing	−0.33	Destabilizing	Uncertain Significance

* Bright green: benign. Bright red: pathogenic. Bolded font variants indicate consistently predicted pathogenicity across the board (light orange shaded cells).

### (A) PLG/R^70^T

This variant is listed as a PDI variant, but it also has uncertain significance in association with hereditary angioedema (HAE) (RCV002493731.1.). In this variant, Arg^70^ located in the AP domain is replaced by Thr (T). This variant may affect the Glu^1^-PLG tight conformation (154). However, predictive analysis of the impact of this variant on PLG sequence and structure is conflicting (Table 4) which may suggest it is partially tolerated and possibly leads to a pathogenic molecular phenotype. Nevertheless, a pathogenic variant does not necessarily result in significant structural perturbation. Further research is required to resolve these contradictions and fully understand the effect of this amino acid substitution.

### (B) PLG/R^153^G

This variant is an example of another rare missense variant of PLG discovered by GWAS. It is associated with increased platelet count, decreased D-dimer concentration, and decreased platelet reactivity (148). The low D-dimer levels are likely due to defective PLG binding to fibrin(ogen) and cells, consistent with reduced fibrinolysis. However, this variant was not associated with a risk for thrombotic disease. Computational analyses consistently predict this variant as damaging and destabilizing (Table 7). Arg^153^ is a key LBS residue (Table 1), and Gly would disrupt the LBS of PLG-K1**.** PLG/R^153^G is ultra-rare but has been detected in European (Finnish and Non-Finnish), African/African Americans, admixed Americans, and South Asians (gnomAD browserv4.0.0). Without functional studies, this variant remains of uncertain significance (VCV001163002.11).

### (C) PLG/D^219^N

Since PLG plays a critical role in inflammation, it has been associated with various diseases with major inflammatory components (155, 156). A whole-genome sequencing study in siblings with cystic fibrosis identified the ultra-rare PDI-associated variant, PLG/D^219^N, in an afflicted patient. This variant was suggested to contribute to the disease by facilitating lung host-pathogen interactions (157). The PLG/D^219^N variants is predicted to be damaging and destabilizing (Table 4). Asp^219^ is a key LBS (Table 1) residue and the substitution with Asn^219^ disrupts the LBS of PLG-K2, thereby affecting the PLG structure by relaxing its closed conformation (32, 118). While PLG/D^219^N is an ultra-rare variant, it has been detected in Middle Eastern, admixed Americans, South Asians, and Europeans (Non-Finnish).

### (D) PLG/K^311^E

(VCV000590291.11) is a clinical variant that produces a functionally distinct PLG phenotype that is pathogenic and has been modeled *in vitro*. Targeted gene panels and disease segregation identified PLG/K^311^E as causative of HAE with normal C1 inhibitor (158). Adding tPA to plasma of patients containing this *PLG* variant leads to an increase in the generation of the vasoactive peptide, bradykinin, and a mechanism for the association of PLG/K^311^E with HAE has been proposed (159). Functional assays of the K^311^E variant support its involvement with the kininogen pathway in hereditary angioedema.

The PLG/K^311^E variant restores the incomplete anionic center of the LBS of PLG-K3 (Table 1). This PLG residue (K^311^) is a Glu in most vertebrates, with the exemption of humans and chimps, where it is a Lys. This may explain why PLG/K^311^E is predicted as tolerated by sequence-based predictive analysis by Polyphen-2 and SIFT (Table 7). However, sequence-based and structure-based stability prediction analysis including MUpro, mCSM, and DynaMut2 consistently show that K^311^E is predicted to be destabilizing. This clinical variant has an autosomal dominant inheritance with variable penetrance (160).

This novel HAE form of the disease is one of various types of HAE with normal C1 inhibitor. While it is unlikely that this variant adopts a more relaxed activation-susceptible conformation, it was suggested that the variant enhances the activation of PLG to plasmin (161). However, experimental evidence shows the activation of this PLG variant is coupled to the contact blood coagulation pathway that produces bradykinin (162). Thus, the mutation in the variant converts PLG/plasmin to an efficient kininogenase, capable of rapidly cleaving kininogen to release bradykinin, leading to an increase in bradykinin concentration and subsequent edema (159).

Interestingly, the PLG/K^311^E variant from plasma has a different glycosylation pattern than normal PLG (163). The authors suggested that the degree of glycosylation in K3-PLG may contribute to the molecular basis of the dysfunction in this mutant, but this needs further evaluation. Glycosylation can affect folding, clearance, and the ability to interact with receptors. It has been previously reported that Glu^1^-PLG, glycoform-I, is easier to activate than Glu^1^-PLG glycoform-II, by both uPA and SK, and it has been suggested that the Asn^289^-linked glycans may influence the interaction of the LBS of K1-PLG with the N-terminal peptide that assists in maintaining the closed conformation of Glu^1^-PLG (164). It is possible that the two PLG glycoforms may have different functionally important conformations (164). Differences in glycosylation affects the relative position of K3-PLG in Glu^1^-PLG (36). The N-linked glycosylation status may also affect the clearance of PLG. Glycosylation appears to influence PLG binding and competition with apo-Lp(a) for LBS' (165). It has been reported that PLG glycoform-I cannot bind to endothelial cells, whereas PLG glycoform-II, as well as non-glycosylated recombinant PLG expressed in *E. coli*, can bind to these cells, and is cleared faster than the fully glycosylated PLG-glycoform I (166).

PLG/K^311^E is an ultra-rare variant and to date it has only been reported in Europeans (Non-Finnish) (gnomAD browserv4.0.0). New guidelines have been introduced in the diagnosis of HAE due to the genetic variability of this spontaneous allergy type syndrome. Novel amino acid variants in several proteins can be involved including some in FXII and other PLG missense variants (167). Genetic screening is important to determine the cause of HAE since it varies in penetrance and will affect further therapeutic development. HAE-PLG, is currently treated with various agents including C1 inhibitor (C1-INH) concentrates, Kallikrein inhibitor, fresh-frozen plasma, injectable ecallantide, and injectable icatibant (168). The mechanism of HAE suggests that antifibrinolytic agents, such as epsilon amino caproic acid (EACA) and tranexamic acid (TXA), may be potential therapeutic options for inhibiting the conversion of PLG/K^311^E to plasmin, thereby halting HAE progression and alleviating symptoms.

### (E) PLG/V^709^E

Another ultra-rare missense variant, PLG/V^709^E, has also been associated with HAE with normal C1 inhibitor (169) and reported as a pathogenic clinical variant (VCV000827591.4). Nonetheless, a patho-mechanism has not been proposed. This substitution may potentially destabilize the salt bridge between residue Lys^708^ of the SP domain and the LBS of the PLG-K2 domain (due to its proximity) which may expose the PLG activation loop, thus resulting in a more activatable PLG. Such a variants may potentially lead to an increased bradykinin concentration and changes in vasopermeability. The PLG/V^709^E variant is predicted to be highly destabilizing to the protein (Table 7). Further studies are needed to clarify the pertinent molecular events.

### (F) PLG/ D^137^N

A recently described PLG missense variant, PLG/D^137^N, from a case report of a young male from Saudi Arabia, is associated with a periodic inflammatory complex syndrome (170). Asp^137^ is an essential residue in the LBS anionic center of K1-PLG (Figure 2A, Table 1) and the Asp^137^Asn substitution is predicted to be damaging (Table 4). This patient was homozygous for PLG/D^137^N and had normal PLG activity, but the PLG antigen concentration was not determined although the patient presented PDI-like clinical symptoms. Unfortunately, this study only included exome sequencing. Limitations of exome sequencing include the potential of missing a significant number of genetic defects (171). This case report further supports the role of PLG in inflammation (170). Recently, a first case of colonic involvement in a congenital plasminogen deficient patient with inflammatory bowel disease further supports the role of PLG in inflammation; PLG antigen levels were low. However, the PLG variant was not identified (172).

### (G) PLG/T^181^A, PLG/G^401^D, PLG/A^475^V, PLG/T^481^M, PLG/A^488^V

These PLG variants were reported to possibly be associated with multiple sclerosis (MS), but their potential role, if any, is uncertain and remains to be confirmed (173). These variants likely facilitate the pathogenesis of MS by increasing the inflammatory response.

**PLG/T^181^A** has uncertain clinical significance (VCV001304561.6) with somewhat conflicting pathogenic predictions (Table 8). It is a low frequency variant found in several ethnicities (Tables 2, 3). It is important to note that Thr^181^, when substituted by Pro, is associated with PDI (Figure 3), and is predicted to be pathogenic (Table 4). This suggests an important role for Thr^181^ in the K2-PLG structure.

**PLG/G^401^D** is a low frequency variant in several ethnicities (Table 2). This variant may have a functional phenotype since it is predicted to be somewhat tolerated with a conflicting pathogenicity (VCV000717878.12) (Table 8). Such a variant could be a risk factor for disease and it is of concern because it is relatively frequent in the population and therefore merits further analysis.

**PLG/A^475^V** is consistently predicted as damaging and destabilizing in our analyses (Table 8) but it is currently categorized as clinically benign. This is likely based on its common appearance in various ethnicities (Table 2) however it may be a risk factor for multifactorial diseases prevalent in populations.

**PLG/T^481^M** is a low frequency variant reported mostly in East Asians (Table 3). This variant has conflicting clinical pathogenicity (VCV001693941.6) and conflictive predictive analyses (Table 8). A potential pathogenic role of PLG/T^481^M is supported by the fact that this variants has been reported as a somatic variants of PLG in primary tissue from various cancers, including central nervous system glioma, large intestinal adenocarcinoma, endometrial carcinoma, esophageal carcinoma, and stomach carcinoma (COSV51981707) (146). This variant requires further study.

**PLG/A^488^V** is an ultra-rare variant of uncertain clinical significance (VCV002173737.1) and is predicted to be pathogenic (Table 7).

It is possible that dysregulation of the PLG/plasmin activation system could contribute to the MS pathology due to its role in inflammatory processes. Interestingly, a study of PLG deficiency in a murine MS model found that a PLG deficiency exacerbates the disease (174). Therefore, although the investigators could not prove a direct connection by segregation analysis, the molecular consequences of these substitutions and a possible role in MS merit further investigation.

### (H) PLG/ K^19^E, PLG/R^234^H, PLG/G^560^R, and PLG/G^693^R

The PLG/K^19^E variant and other relatively abundant variants of concern found in PDI or PDII, *viz.,* R^234^H (Tables 2, 4) and PLG/G^693^R (Tables 2, 3, 6, 8), have been reported in patients with atypical hemolytic uremic syndrome (AHUS) (175).

**PLG/R^234^H** is consistently predicted to be pathogenic and destabilizing (Table 4). Nonetheless, this variant is currently listed as likely benign (VCV000724207.6). Inconsistencies of clinical classification with predictions for many PD variants may be due to variable penetrance of phenotypes and very low abundance of the variants.

**PLG/G^693^R** is predicted pathogenic and destabilizing (Table 6) with an uncertain significance and leads to PDI and PDII in a compound heterozygous state with PLG/K^19^E (93, 123). Interestingly, G^693^R is relatively abundant in Native Americans (Table 3) and in the Ashkenazi Jewish population (Table 2).

**PLG/G^560^R**, is associated with PDI, atypical hemolytic-uremic syndrome and HAE (176). This variant is predicted to be harmful and destabilizing (Table 4) but remains as having uncertain clinical significance (VCV000988227.3). Further studies are required to confirm whether the PLG/plasmin system is part of AHUS pathogenesis. Thus, the involvement of PLG/K^19^E, PLG/R^234^H, PLG/G^560^R, and PLG/G^693^R in AHUS should not be ruled out at this point.

### (I) PLG/D^453^N

The most abundant PLG missense variant in the world is PLG/D^453^N, which is polymorphic (MAF% ≥5) in most ethnic groups, except for East Asians where it is considered to be a rare variant (Tables 2, 3). Prediction analysis indicates that the polymorphic PLG/D^453^N is benign and tolerated (Table 8) but an association with disease, especially when other variants are present, cannot be ruled out. When combined with some other PLG missense deleterious variants, polymorphic PLG/D^453^N seems to produce the PDI phenotype (177). In two heterozygous patients with LigC carrying a PLG/G^199^V variants in K2-PLG with heterozygous PLG/D^453^N, the additional presence of PLG/D^453^N was sufficient to produce the severe PDI phenotype (103). Numerous PDI case reports have shown that affected patients carry PLG/K^19^E and/or PLG/D^453^N, in addition to rare PLG variants. It is important to note that PLG/D^453^N has also been associated with *otitis media* (113), which has been found to occur spontaneously in PLG-deficient mice (178). Asp^453^ is not a highly conserved residue, as it is substituted with Asn in many primates and a Ser in mouse PLG.

PLG/D^453^N has been proposed to be a genetic risk factor for invasive *Aspergillosis* (IA) infections (179). Most risk factors for IA involve immune system components. The relationship of the fibrinolytic system with the immune system has been more recently highlighted (180). In a separate study, it was shown that cell surface enolase from *A. fumigatus* binds plasma-derived PLG with a K_D_ of 530 nM for WT-PLG (181). This surface bound PLG can be activated to plasmin to facilitate pathogen invasion (181). A single amino acid substitution in mPg, *viz.,* PLG/G^91^S, enhances the murine K1-LBS and confers susceptibility to *A. fumigatus* in an immunosuppressed murine disease model, thereby supporting a critical role for PLG in susceptibility to IA (179). The role of polymorphic PLG/D^453^N in its binding and activation in IA needs further examination. Binding assays to compare PLG/Asp^453^ and PLG/Asn^453^ variants to PLG receptors of this pathogen could facilitate determination of its role. Tolerated overabundant variants like PLG/Asn^453^ may exhibit a somewhat different phenotype or acquire novel binding partners.

### (J) PLG/R^504^W

PLG/R^504^W is an important worldwide variant with a total MAF% of 1.19% (Table 2). It is important in multiple ethnicities (Tables 2, 3). This variant has been associated with lower plasma PLG concentrations (146) and it is clearly a heritable risk factor that may contribute to the variation in PLG levels in some individuals and populations. This variant is currently labeled as benign (VCV000770367.9), yet it is predicted to destabilize the protein (Table 8). Recently, a PLG/R^504^W homozygous individual, identified by using the Qatar Biobank, was found to have very low levels of PLG and angiostatin but normal levels of active plasmin. Moreover, this individual presented with enhanced thrombosis that required warfarin intake (182). Nevertheless, PLG/R^504^W remains as a conflicting variant.

### (K) PLG/R^471^Q and PLG/I^663^N

The pathogenicity of two relatively abundant PLG missense variants: PLG/R^471^Q and PLG/I^663^N (Tables 2, 3) remains uncertain. From these, PLG/R^471^Q (149) is a clinical variant (VCV000076224.11) with conflicting pathogenicity. It has been associated with various diseases like PDI, thrombocytopenia, abnormal bleeding, and deep vein thrombosis. It is most prevalent in Europeans and is predicted as deleterious. Therefore, it is a low frequency variant of concern in various ethnic groups, mainly European and in some of the Americas. Interestingly, the R^471^Q allele can lower PLG levels to facilitate the development of PDI (113). The missense variant PLG/I^663^N is also consistently predicted to be destabilizing and pathogenic (Table 8). It is mostly limited to Europeans as a low frequency variant of concern (Tables 2, 3). The potential contribution of PLG/I^663^N to *otitis media*, PDI, and deep venous thrombosis is uncertain (VCV000692203.10).

Other PLG variants predicted to be damaging but not as yet correlated with any pathogenic effect include PLG/E^38^K, PLG/R^70^K, PLG/D^175^V, PLG/R^242^H, PLG/R^389^W, and PLG/S^441^R (Table 8). PLG/E^38^K, PLG/R^389^W, and PLG/S^441^R are prevalent among African/American populations. Notably, the phenotypic consequences of carrying homozygous or compound heterozygous of these various common PLG missense variants have not been investigated.

**Figure 5:**
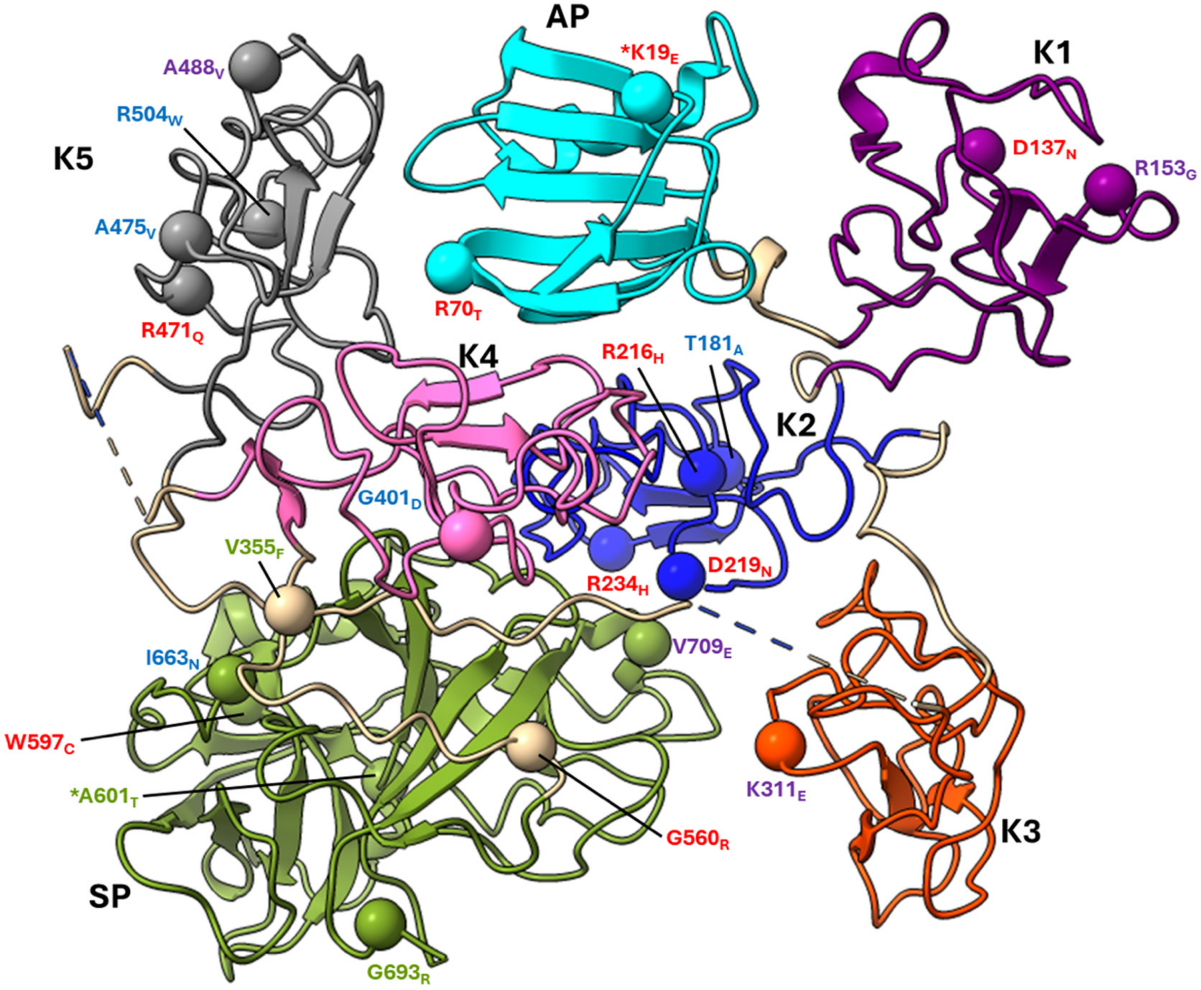
Placement of several, mostly pathogenic, PLG variants within the PLG x-ray crystal structure (PDB ID, 4DUR). Missense variants are designated red in PDI and green in PDII (green). Major PLG missense variants in the population are presented in blue and other pathogenic variants are labeled purple. The asterisk in *K^19^E and *A^601^T indicates that these two variants are also relatively abundant. Color codes for PLG domains include cyan for activation peptide (AP), purple for kringle 1 (K1), blue for kringle 2 (K2), orange for kringle 3 (K3), pink for kringle 4 (K4), grey for kringle 5 (K5), green for the serine protease (SP) domain, and tan for interdomain loops. This collection of variants is associated, or possibly associated, with several disorders. Table 9 lists these variants and corresponding full protein numbering as well as disease association.

To conclude our analysis, Figure 5 illustrates a collection of mostly consistently pathogenic PLG missense variants and maps them in the PLG x-ray structure. This figure summarizes the position of such collection of most relevant variants that are also listed in Table 9. These variants relate to specific pathologies including PD but also diseases other than PD that are either associated or suspected to be associated with the respective PLG variants.

**Table 9:** Collection with PLG missense variants and their association (or potential association) to various diseases in addition to, or other than, PD*.

Mature protein variant	Full protein variant	Potential or associated disorder	Current clinical status
K19E	p.K38E	PDI, LigC, susceptibility to Otitis Media	Conflictive pathogenicity
R70T	p.R89T	PDI, HAE with normal C1 inhibitor	Uncertain significance
D137N	p.D156N	PDI, Periodic Inflammatory Complex Syndrome	Not reported
R153G	p.R172G	Increased platelet count, D-dimer concentration, and platelet reactivity	Uncertain significance
T181A	p.T200A	Multiple Sclerosis	Uncertain significance
R216H	p.R235H	PDI, LigC	Pathogenic
D219N	p.D238N	PDI, Cystic fibrosis	Risk Factor
R234H	p.R253H	PDI, Atypical Hemolytic Uremic Syndrome	Likely Benign
K311E	p.K330E	HAE with normal C1 inhibitor	Pathogenic
V355F	p.V374F	PDII, predisposition to thrombosis	Pathogenic
G401D	p.R490Q	Multiple Sclerosis	Conflicting Pathogenicity
R471Q	p.R490Q	PDI, thrombocytopenia, abnormal bleeding, and Deep Vein Thrombosis	Conflicting Pathogenicity
A475V	p.A494V	Multiple Sclerosis	Benign
A488V	p.A507V	Multiple Sclerosis	Uncertain significance
R504W	p.R523W	Lower plasma PLG concentrations, low Angiostatin	Likely Benign
G560R	p.G579R	PDI, Atypical Hemolytic-Uremic Syndrome and HAE	Uncertain significance
W597C	p.W616C	PDI, LigC	Pathogenic
A601T	p.A620T	PDII, predisposition to Thrombosis	Conflictive pathogenicity
I663N	p.I682N	Otitis media, PDI, and Deep Venous Thrombosis	Conflicting Pathogenicity
G693R	p.G712R	PDII, Atypical Hemolytic Uremic Syndrome	Uncertain significance
V709E	p.V728E	HAE with normal C1 inhibitor	Pathogenic

* All variants have been discussed in the text and are illustrated in Figure 4.

Overall, we find that predictions resulting from tools based on protein structure including mCSM and DynaMut2 were more consistent with the reported PLG phenotypes. Sequence-based predictions, mostly based on sequence conservation like Polyphen-2 and SIFT, were not always consistent with reported phenotypes. Notably, the sequence-based MUpro tool was found to be very consistent with described phenotypes. Computational tools that rely on calculations of the resulting change in folding free energy (ΔΔ*G*) caused by amino acid substitutions including MUpro, mCSM and DynaMut2 were much more consistent with detected phenotypes. Stability-based predictions have been found to be very reliable in detecting potential, disease associated, amino acid substitutions (183).

Not all destabilizing amino acid substitutions in PLG may lead to misfolding, aggregation and increased clearance. It is possible that some of the destabilizing substitutions associated with PDI will lead to increase clearance, while others may impair both binding to molecular partners as well as increased clearance. Example of the latter may include substitutions that perturb the LBS domains. This is supported by a majority of PDI variants located in the kringle domains. The compromising of the very conserved kringle domains may also lead to misfolding and faster clearance. Some other destabilizing amino acid substitutions in PLG, including those for variants found in major populations, may contribute to human disorders by partially destabilizing the tertiary structure of the protein and leading to novel molecular/functional phenotypes.

## PLG missense variants and cancer

To date, no *PLG* germline genetic variants have been reported to directly lead to cancer. However, a potential role of *PLG* missense somatic variants in cancer (and other diseases) should not be ruled out. The PLG/plasmin system plays a fundamental role in the migration of malignant cells and metastasis in solid tumors, and it is directly involved in the activation of matrix metalloproteases (184, 185). Pericellular plasmin facilitates the invasion process and PLG receptors are found on the surface of most tumors. The expression of PLG receptors can be used for cancer prognosis and survival (186). Regulation of the PLG/plasmin system can result in the stimulation or suppression of cancer (187). Whole genome/exome database and computational and experimental analyses can facilitate identification of driver genes and to determine the role of missense variants in cancer (2). *PLG* is a cancer-related gene based on experiments involving insertional mutagenesis in mice, but it is not considered to be a cancer-driver gene. Somatic mutations accumulate during malignant transformation (188) and other complex diseases (189). Mutations that directly lead to a tumor proliferative advantage are considered driver mutations but those account for a very low (3%) proportion of observed genetic aberrations in cancer. *PLG* somatic genetic mutations found in tumors are curated by the catalogue of somatic mutation in cancer (COSMIC) among other databases. Hundreds of somatic variants of *PLG* have been reported and catalogued from diverse tumors. The most common type of *PLG* variants found in tumor samples curated by COSMIC are variants in the protein coding sequence, with missense variants representing 45% of the total.

Whereas missense mutations are frequently found in malignancy, their role is not easy to predict (190). To date, no *PLG* somatic mutations have been identified as a driver cancer mutations Many of the *PLG* missense variants discussed in this review have been also detected as somatic variants in diverse tumors but they are predicted as passenger mutations using the FATHM cancer algorithm prediction tool (https://fathmm.biocompute.org.uk) (191). The role of passenger mutations in cancer however is currently poorly understood but the concept of such genes playing important roles in malignancy evolution is increasingly supported (192). Passenger mutations constitute most (>97%) of the somatic mutations present in tumors. Some passenger mutations can become established and become part of the clonal progression of a tumor and may affect the tumor phenotype, *e.g.,* drug susceptibility and antigenicity. Passenger mutations can also affect tumor growth properties or even lead to tumor regression. These mutations can also be used to classify tumor type and help determine the origin and history of metastatic lesions by serving as a molecular clock on cancer evolution (193). The collective burden caused by passenger mutations can help to explain the progression of cancer not explainable by driver genes alone (194). The tumor type, and its evolution and prognosis, can be influenced by the accumulation of somatic mutations (195).

A systematic study of the progression of missense somatic mutations of *PLG* and their potential role in cancer evolution is lacking. It is possible that particularly pathogenic PLG missense variants, like those that cause PDI and PDII, will impair tumor progression and will not be selected in the clonal expansion of a tumor. But those that facilitate PLG binding to cellular receptors or enhance PLG activation would possibly promote malignancy and may represent important therapeutic targets. Two critical parameters, sometimes missing from tumor databases, include confirmation of somatic vs. germline origin of mutations and zygosity. These gaps can hinder a comprehensive understanding of the role of missense variants in tumor evolution.

*PLG* passenger somatic missense variants may be involved in the cancer mutational progression landscape and represents a potentially important point to investigate further.

Missense variants in *PLG* may also play an indirect role in cancer by affecting the type of posttranslational modifications that occur. Phosphorylation is a reported post-translational modification that can lead to many types of cancer (196, 197). Recently residue PLG/Tyr^92^ present in PLG-K1 was flagged by a novel bioinformatic proteomics and cancer co-clustering tool as a potentially relevant cancer-associated phosphorylation site in PLG (196).

## PLG missense mutations and COVID-19

The pandemic of COVID-19 emphasized how diverse host genetic differences at the individual level can affect the outcome of the disease (198). For instance, COVID-19 presenting with an inflammatory response can become a systemic thrombotic disease in susceptible individuals and, as such, the circulating PLG concentration is a current new key parameter obtained from patients on hospital admission (199). PLG and plasmin are key participants in homeostasis and other pathological states (200). These proteins play critical and complex roles in COVID-19 pathogenesis (201) and their dysregulation can influence the outcome of COVID-19 patients. Recent studies revealed that low PLG levels were the most significant prognosticators of death in COVID-19 patients (199, 202), being also associated with higher inflammation parameters. Potentially, patients with reduced PLG may be more susceptible to poorer outcomes in COVID-19 and other inflammatory diseases. In these cases, treating patients with PLG during the acute phase of the disease has been found to be beneficial (201).

The heterogeneity of susceptibility and outcomes to COVID-19 can be affected by genetic variants in the population (198). Ethnic genetic variants of PLG as potential determinants of heterogeneity in response to COVID-19 has been recently suggested (203). The importance of PLG in COVID-19 accentuates a potential clinical significance of polymorphic PLG carried in different populations toward this and other diseases.

Atypically low concentrations of PLG may be contributing risk factors for this and other diseases. These PD states can be genetic in combination with single or combined PLG polymorphisms or acquired during the disease state. In any event, studies relating to the PLG genetic complexities with diseases, such as COVID-19, are lacking.

## Studies with PLG-deficient mice

While not the focus of this review, the generation of PLG^−/−^ mice allowed unprecedented studies of the role of PLG *in vivo* at multiple levels. Mice with a total deficiency of PLG (mouse PLG^−/−^) have severe lifelong challenges, including deficiencies in vascular wound healing (204) and vascular remodeling after arterial injury (205), as well as venous and arterial thrombosis (134), despite the fact that PLG is not the only fibrin degrading enzyme in the vasculature (89, 90). A study assessing the development of LigC in PLG^−/−^ mice demonstrated an equivalent phenotype to that observed in PLG-deficient humans. However, mice deficient for both PLG and fibrinogen did not develop ligneous conjunctivitis thereby linking PLG/plasmin-mediated clearance of fibrin as a regulatory mechanism for this disease (135). Endothelial cells from mice deficient in PAs or mouse PLG can penetrate fibrin barriers with metalloproteinases acting as fibrinolysins (206). Moreover, endothelial cells ensure and contribute to vascular system patency by producing fibrinolytic activity through MMPs in the absence of PLG (207).

A mouse PLG knock-in carrying the homozygous mouse PLG/A^603^T allele did not show an increased susceptibility to thrombosis, as compared to WT mice when challenged in experimental thrombotic models (129). Unlike the reported PLG deficiencies in humans, the PLG^−/−^ mouse model, wherein PLG is totally absent, shows a fundamental need for PLG for a healthy life (90). Thus, it is reasonable to extrapolate that the complete absence of plasmin in humans will be damaging and having low PLG activity could increase susceptibility to thrombosis after a challenge (137, 208, 209).

## Conclusions and perspectives

The most well-known PLG variants are a group of rare pathogenic missense variants that lead to PDI and PDII and are described in family case reports. The true prevalence of these variants is unclear, and they may constitute a disease risk in heterozygous carriers. In addition to the codominant PLG/D^453^N, approximately ten other PLG missense variants are rather abundant in various world genetic ancestries. Some of them have disease association and predicted pathogenicity, including PLG/K^19^E and PLG/A^601^T, which associate with PDI and PDII, respectively. The abundant PLG/R^504^W variant that lowers Pg levels, and several other prevalent and predicted pathogenic variants, such as PLG/R^242^H, PLG/R^471^Q, PLG/A^475^V, and PLG/T^181^A, most likely contribute to complex disorders and deserve further attention. These findings are consistent with PLG/plasmin having important involvement not only in fibrinolysis, but also in wound healing, inflammation, immune response, and pathogen invasion. The PLG concentration and activity vary considerably among the global population which could in part be a consequence of carrying some of those PLG alleles. PLG/K^19^E and PLG/A^601^T, initially described more than 20 years ago, are still relevant today when individual heterogeneity and differential susceptibility to disease has become increasingly evident. The different susceptibilities to the progression of COVID-19 require clearer understanding of unique genetic background of critical parameters, such as the PLG levels and the PLG activation potentials. We herein review how ethnic backgrounds influence the nature of the PLG variants carried, and what regions in the world are more susceptible to these genetic diseases. This knowledge is relevant when designing global therapeutic and prophylactic interventions. Several other rare PLG missense variants have been associated with disease by GWAS. It is thought that many complex diseases can result from additive effects of even moderate pathogenicity from individual missense variants. The important role of PLG in inflammation and allergy is confirmed by the direct connection of the PLG/K^311^E variant with HAE with normal C1 inhibitor. The present review highlights how PLG activity and concentration can be much lower than originally expected. Variations in the PLG level and activity in plasma and extravascular tissues can have severe consequences in combination with other factors. This comprehensive view of PLG missense variants and disease association in a global context is relevant to epidemiology of diseases. The information discussed herein can impact personalized medicine, *e.g.,* a knowledge of specific variants and associated pathology can help in diagnosis (development of targeted diagnostic kits) and tailored treatment strategies (development of novel therapeutics), optimizing outcome of PLG associated disorders and minimizing adverse reactions. Moreover, it is useful for the development of prophylactic strategies in different world populations carrying certain variants. The use of IEF for detecting PLG alleles can still be of use for paternity tests where no molecular biology methods are feasible. Also, the information provided in this review is relevant for genetic counselling and risk assessment. For instance, identifying high-risk populations can lead to early interventions and monitoring, potentially preventing disease progression. Deciphering molecular mechanisms of PLG-related genetic diseases will continue to reveal the *in vivo* significance of the PLG/plasmin system. Our intention is to bring a global perspective and awareness of PLG heterogeneity to the population and their susceptibilities to disease beyond fibrinolysis. Awareness of the clinical significance and disease risk of PLG polymorphisms will provide useful information that will assist development of new therapies for a number of diseases in which PLG plays a role.
